# TRPV1 temperature activation is specifically sensitive to strong decreases in amino acid hydrophobicity

**DOI:** 10.1038/s41598-017-00636-4

**Published:** 2017-04-03

**Authors:** Jason O. Sosa-Pagán, Edwin S. Iversen, Jörg Grandl

**Affiliations:** 1 Department of Neurobiology, Duke University Medical Center, Durham, NC 27710 USA; 2 Department of Statistical Science, Duke University, Durham, NC 27710 USA

## Abstract

Several transient receptor potential (TRP) ion channels can be directly activated by hot or cold temperature with high sensitivity. However, the structures and molecular mechanism giving rise to their high temperature sensitivity are not fully understood. One hypothesized mechanism assumes that temperature activation is driven by the exposure of hydrophobic residues to solvent. This mechanism further predicts that residues are exposed to solvent in a coordinated fashion, but without necessarily being located in close proximity to each other. However, there is little experimental evidence supporting this mechanism in TRP channels. Here, we combined high-throughput mutagenesis, functional screening, and deep sequencing to identify mutations from a total of ~7,300 TRPV1 random mutant clones. We found that strong decreases in hydrophobicity of amino acids are better tolerated for activation by capsaicin than for activation by hot temperature, suggesting that strong hydrophobicity might be specifically required for temperature activation. Altogether, our work provides initial correlative support for a previously hypothesized temperature mechanism in TRP ion channels.

## Introduction

Transient Receptor Potential (TRP) ion channels form a family of non-selective cation channels that are activated by a wide variety of chemical and physical stimuli^1^. For example, the first identified and biophysically most well-characterized mammalian TRP channel TRPV1 is activated by capsaicin (an alkaloid present in hot chili peppers), voltage, acidic extracellular and basic intracellular pH, the double-knot toxin (DkTx) found in a tarantula venom, and hot temperatures^2–6^. TRPV1 is expressed in small diameter dorsal root ganglion neurons and functions as the principal sensor of several of these stimuli. Thus, mice lacking TRPV1 have deficiencies in sensing noxious hot temperature, exhibit reduced thermal hyperalgesia, and are insensitive to capsaicin^7, 8^. While the mechanism of ion channel activation by voltage or chemicals is understood in principle, the mechanism of activation by temperature is unknown^6, 9–16^. The fact that several TRP channels can be activated by temperature in cell-detached patches, and in artificial bilayers of various lipid compositions with sub-millisecond latency strongly suggests that the activation mechanism is directly mediated by the membrane bilayer and the channel, and not indirectly by second messengers^17–19^. However, it is not definitively known whether temperature activation occurs through intrinsic high temperature sensitivity of the channel protein or through modulation of physical properties of the bilayer. Although lipid dislocation has been identified as a possible allosteric regulator of TRPV1 activation, the former possibility seems more likely, since fundamental thermodynamic properties, such as temperature activation directionality (heat vs. cold), threshold, and sensitivity (Q_10_ value) are determined by the protein and not the bilayer composition^20–23^.

Generally, ion channels have a modular architecture, with distinct structures for specific channel functions, such as voltage sensors, ligand binding pockets, or pore domains, suggesting the existence of a distinct temperature sensor domain^14–16, 24^. TRP channels were assumed to follow this paradigm, since they have high structural similarity to voltage-gated potassium channels (K_v_)^25^. This concept is also supported by the fact that protein secondary structures differ substantially in thermodynamic stability, which can lead to highly localized effects of temperature on protein conformation^26, 27^. Thus, numerous previous studies hypothesized and aimed to identify a ‘temperature sensor domain’ by amino acid deletion, chimeric constructs, analysis of channel isoforms, or mutagenesis. In three separate studies, deletion of the proximal N-terminus (M1-P109) and the distal C-terminus (N765-K838) of TRPV1, 15 residues between S5 and the pore helix loop (T612-S626) of TRPV1, or the complete N-terminus of human TRPA1 did not affect temperature activation, demonstrating that not all domains contribute to temperature gating^18, 28, 29^. Chimeric studies between TRPV1 and TRPV2, as well as comparisons of TRPA1 orthologues and *Drosophila* isoforms, have identified ankyrin repeats, and domains within the N-terminus as modulators of temperature threshold and thermal sensitivity coefficient (Q_10_)^21, 30–33^. Unbiased mutagenesis screens identified residues in the outer pore domains of TRPV1 (N628, N652, and N653), TRPV3 (I644, N647, and Y661), and TRPA1 (G878V) to be specifically required for thermal, but not for chemical activation^22, 34, 35^. In TRPV1 these mutations destabilize the open state and thereby reduce overall temperature sensitivity^34^. Finally, our lab identified single point mutations in ankyrin repeat 6 (S250, M258, D261) of mouse TRPA1 that individually change temperature activation directionality (heat vs. cold), which we interpreted as originating from a change in the coupling of temperature sensing to channel gating, rather than actual temperature sensing^20^. Collectively, these studies implicate many domains in some aspect of temperature activation, which raises the possibility that a temperature sensing structure is not localized in a single coherent amino acid sequence, but instead formed by tertiary structure or in the interphase between subunits of the tetramer. Unfortunately, the available high-resolution structures of TRPV1, TRPV2, and TRPA1 were obtained in the presence or absence of chemical agonists and not at different temperatures thus not providing clear structural correlates of temperature activation^29, 36–39^. Alternatively, domains responsible for high temperature sensitivity in TRP channels might be entirely distributed throughout the protein. Exactly this dispersed localization of structures is consistent with a mechanism assuming that large changes in heat capacity (∆C_p_), mediated by the coordinated exposure of hydrophobic residues to solvent, may drive cold and heat activation^40^. Applying this thermodynamic principle, a temperature insensitive K_v_ channel was engineered to become cold or heat sensitive^41^. However, while this bottom-up approach is a strong conceptual demonstration, it remains unclear whether the same mechanism underlies temperature activation of TRP channels. While there is currently no experimental evidence for this mechanism, it makes the strong prediction that hydrophobicity of residues drives temperature activation.

Here, we used deep mutational scanning to correlate the effect of 287 and 248 mutations to TRPV1 channel activation by temperature or capsaicin, respectively. We found that strong decreases in hydrophobicity of TRPV1 amino acids are better tolerated for activation by capsaicin than for activation by hot temperature. This provides initial correlative support for amino acid hydrophobicity being important for temperature activation of a TRP channel.

## Results

### Ultra-Deep Sequencing Identifies 535 Functionally Characterized Mutations

**Figure 1:**
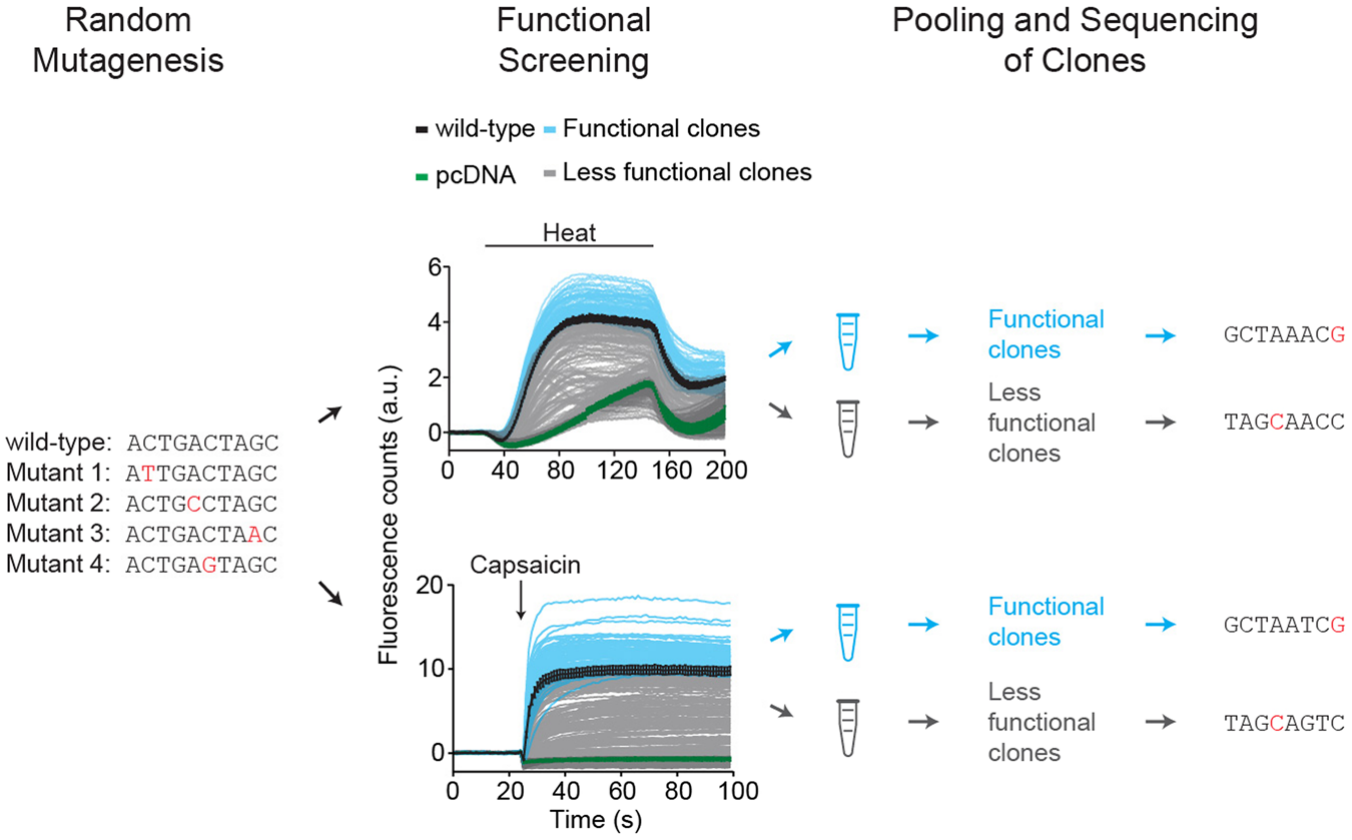
Schematic representation of experimental approach. Illustration of random mutagenesis, calcium-based functional screening, pooling of clones with identical functionality, and deep sequencing of pooled clones. Fluorescence responses of 94 mutant clones (4 responses per clone) upon heat (top panel) or capsaicin (bottom panel) stimulation. Wild-type TRPV1 (black), pcDNA (green), ‘functional’ clones (blue), and ‘less functional’ clones (gray). Wild-type TRPV1 and pcDNA are average responses of n = 4 wells. Error bars represent s.d.

We made use of ~8,500 randomly mutated clones of rat TRPV1 that were previously functionally characterized for temperature and capsaicin activation with a fluorescence based calcium influx assay^34^. We compared heat-evoked (25 °C to 45 °C) responses of each clone to wild-type TRPV1 and pcDNA. We then selected all clones that showed normal activation by temperature or capsaicin as compared to wild-type controls on the same assay plate (see Methods) (Fig. 1). We further refer to these clones as temperature ‘functional’ and capsaicin ‘functional’, respectively. Clones were tested in quadruplets, and each of them was only considered for further analysis if at least three of the four quadruplets were identically assigned. This resulted in a total of ~1,795 categorized temperature ‘functional’ and ~1,479 capsaicin ‘functional’ clones.

**Figure 2:**
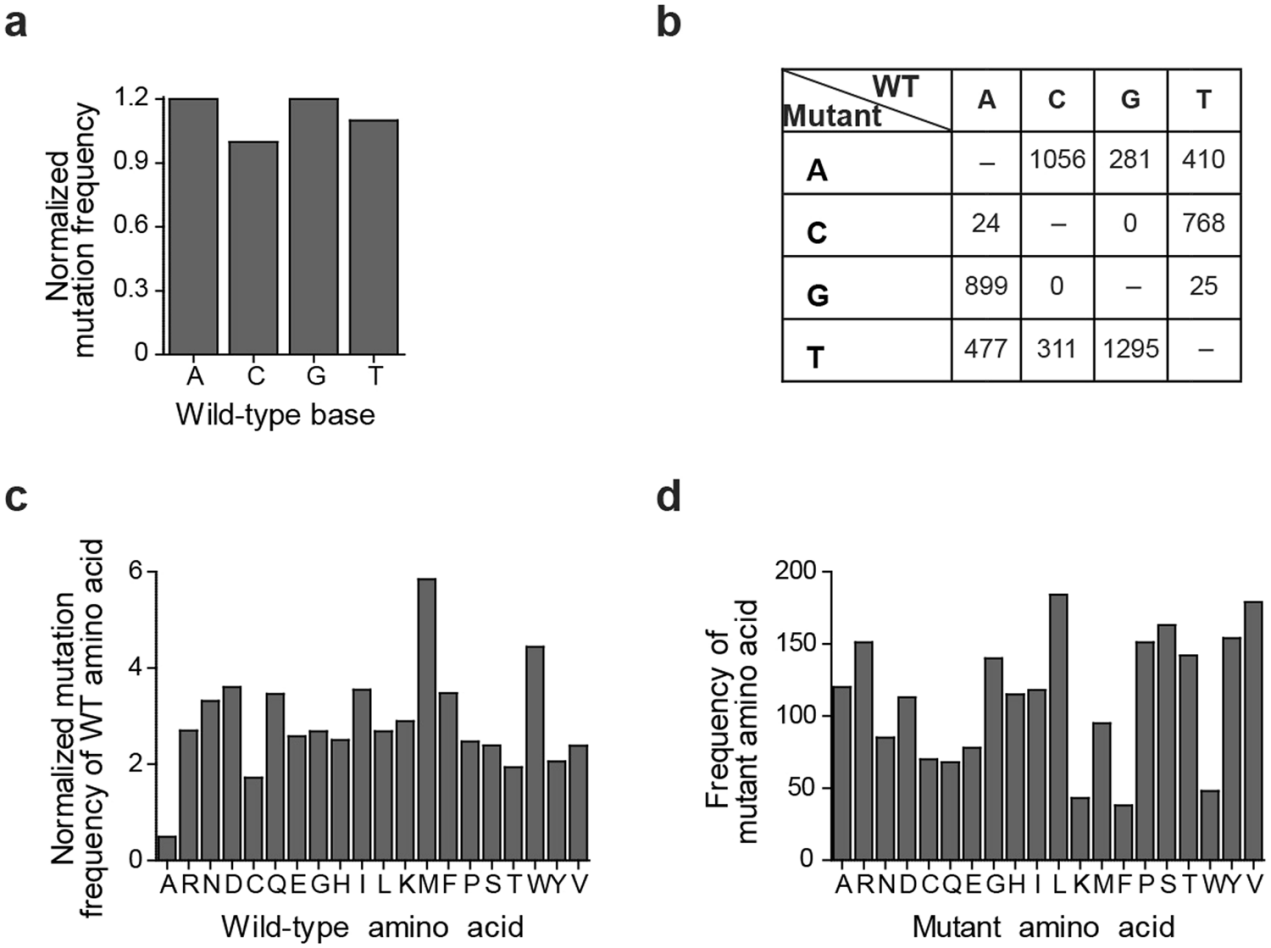
Numbers of base pairs mutations of TRPV1 identified by sequencing clones stimulated with temperature and capsaicin. (**a**) Normalized mutation frequency for wild-type bases for the temperature and capsaicin screened libraries. (**b**) Numbers and identity of base pair mutations in correlation to the wild-type base. (**c**) Number of times wild-type amino acids were mutated. (**d**) Frequency of mutant amino acid.

To identify mutations that do not affect temperature or capsaicin responses, temperature ‘functional’ and capsaicin ‘functional’ clones were pooled and sequenced in a single lane with 150 paired-end read length in an illumina HiSeq 2500 sequencer. In order to explore the sequence diversity of the mutant library we sequenced the clones that were classified as temperature ‘less functional’ (~5,505) and capsaicin ‘less functional’ (~5,821) in the same lane as ‘functional’ clones (see Methods). We obtained an average coverage of more than 700,000x, which allowed us to detect low frequency (~1 in 600 bp) mutations. We determined the variant call threshold by adjusting the error rate of a binomial distribution, postulating that no stop codons should be present before the pore domain (Q560) of the ion channel in the capsaicin ‘functional’ category with a confidence level of 95%. The determined error rate (0.0016) was then applied to all sequenced mutations (see Methods). Analysis of all base pair mutations showed that wild-type bases were mutated at nearly equal frequencies (A and G = 1.2, C = 1.0, and T = 1.1) with a minor bias of transversions T_v_ (purine to pyrimidine and pyrimidine to purine) compared to transitions T_s_ (purine to purine and pyrimidine to pyrimidine), with a ratio T_s_/T_v_ = 0.7, which is consistent with the error-prone PCR method we used (Fig. 2a and b). Moreover, on a base pair level the library explored a large fraction (73%) of the attainable sequence space (see Methods). At the amino acid level most wild-type residues were mutated at similar frequencies (~2–4x), with the exception of less frequently mutated alanines (~0.5x) and more frequently mutated methionines (~6x) and tryptophanes (~5x) (Fig. 2c). Due to the varying occurrence of wild-type amino acids and the degeneration of the genetic code the number of introduced mutant residues differed more widely (~30–180 per amino acid) (Fig. 2d). Altogether, the library covered ~14% of the total amino acid sequence space.

We identified 287 temperature ‘functional’ and 248 capsaicin ‘functional’ amino acid mutations and 1,720 additional ‘less functional’ amino acid mutations for a total of 2,255 amino acid mutations in the whole library covering 80.1% (671 of 838) of all amino acids of TRPV1 (Fig. 2c and Supplementary Tables S1 and S2). Since various processes that are unrelated to the mechanisms of temperature and capsaicin activation, such as protein misfolding, inefficient channel trafficking, or altered ion permeation, can all compromise channel function, we focused exclusively on temperature ‘functional’ and capsaicin ‘functional’ mutations and analyzed the remaining 1,720 amino acid mutations only to characterize the diversity of the library. We reasoned that although ‘functional’ clones may carry more than one mutation, it is unlikely that any of these is compromising function by itself and only leading to a wild-type like phenotype because it is rescued by a second mutation, thus making a misclassification of mutations from ‘functional’ clones highly unlikely.

Altogether ~39% of the mutated residues were mutated once, ~30% mutated twice, and ~10% mutated more than two times. Furthermore, we found that 49.1% of temperature ‘functional’ mutations were also capsaicin ‘functional’. Taken together, our approach of combining random mutagenesis and high-throughput functional screening with next generation sequencing thus identified an unprecedented number of mutations that did not affect temperature or capsaicin activation of TRPV1.

### Most Structural Domains of TRPV1 are Tolerant to Mutations

**Figure 3:**
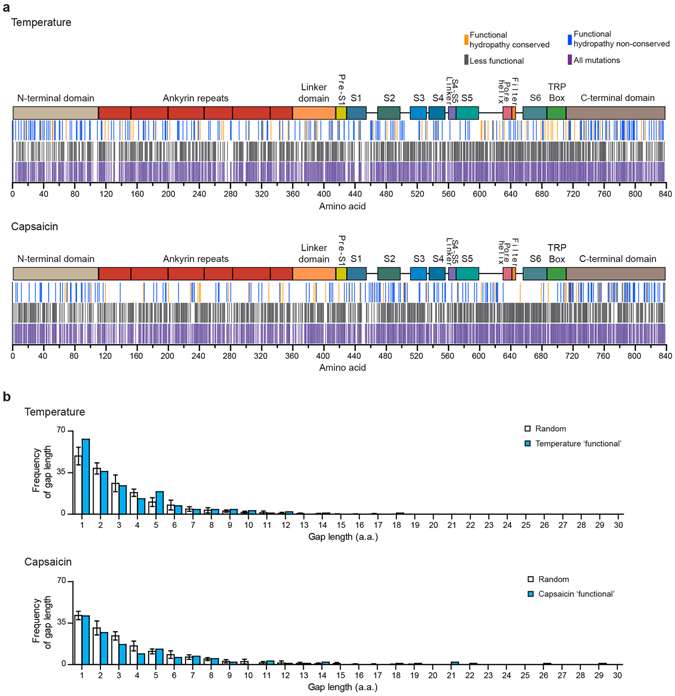
Location of identified mutations. (**a**) Illustration of the location of identified mutations screened with temperature (top panel) or capsaicin (bottom panel) for ‘functional’ clones (blue and yellow), ‘less functional’ clones (gray) or all identified mutations pooled together (purple). In yellow are ‘functional’ mutations that conserved wild-type amino acid hydropathy. In blue are ‘functional’ mutations that did not conserve amino acid hydropathy. Mutations were considered hydropathy-conserving if the Δhydropathy (hydropathy_mutant_ − hydropathy_WT_) = ±0.6. Mutations outside of that range are considered non-conserving. Each bar indicates one single mutation and boxes on top illustrate the structural domains of TRPV1. (**b**) Histogram of frequencies of gap lengths between mutations of temperature (top) or capsaicin (bottom) ‘functional’ category and gap lengths within ten random distributions of 287 (temperature, top) or 248 (capsaicin, bottom) mutations in 838 positions. Error bars are SEM.

Due to the low throughput of site-directed mutagenesis and high costs of Sanger sequencing, knowledge about the effects point mutations have on temperature and capsaicin activation is limited to only a small number of amino acids^42^. Our data from 287 temperature ‘functional’ and 248 capsaicin ‘functional’ mutations might thus point towards large structures, domains, or motifs for temperature and capsaicin activation. Specifically, we hypothesized that residues/domains important for temperature or capsaicin activation should be sensitive to mutations, whereas residues/domains not important should be more tolerant. By mapping all ‘functional’ mutations onto the primary amino acid sequence, we found that for both stimuli, mutations were distributed throughout the entire protein sequence (Fig. 3a). Particularly, the fact that these mutations are distributed throughout the primary sequence contrasts with the idea that within the TRPV1 ion channel coherent domains or sequence motifs mediate channel activation by temperature or capsaicin. To analyze if the introduced mutations were similar to wild-type amino acids we quantified the change in amino acid hydropathy (hydropathy_mutant_ − hydropathy_wild-type_) using the Kyte-Doolittle scale and found that out of 287 temperature-characterized mutations 80 were hydropathy-conserving and 207 were non-conserving mutations (Fig. 3a)^43^. For capsaicin 59 of the 248 mutations were hydropathy-conserving, whereas 189 were non-conserving. To test quantitatively if the temperature or capsaicin ‘functional’ mutations are distributed randomly or instead reveal domains that are sensitive to mutations, we generated a histogram of the frequencies of gap lengths between temperature or capsaicin ‘functional’ mutations and found that it was nearly identical to a distribution of ten randomly mutated sequences (Fig. 3b). We also mapped all temperature and capsaicin ‘functional’ mutations onto the high-resolution structure of TRPV1, but did not find obvious regions that are enriched or devoid of mutations (Supplementary Fig. S1). Altogether, our data do not point towards large and coherent structural domains that are intolerant to mutations.

### Decrease in Amino Acid Hydrophobicity is Less Frequently Tolerated for Activation by Temperature than for Activation by Capsaicin

**Figure 4:**
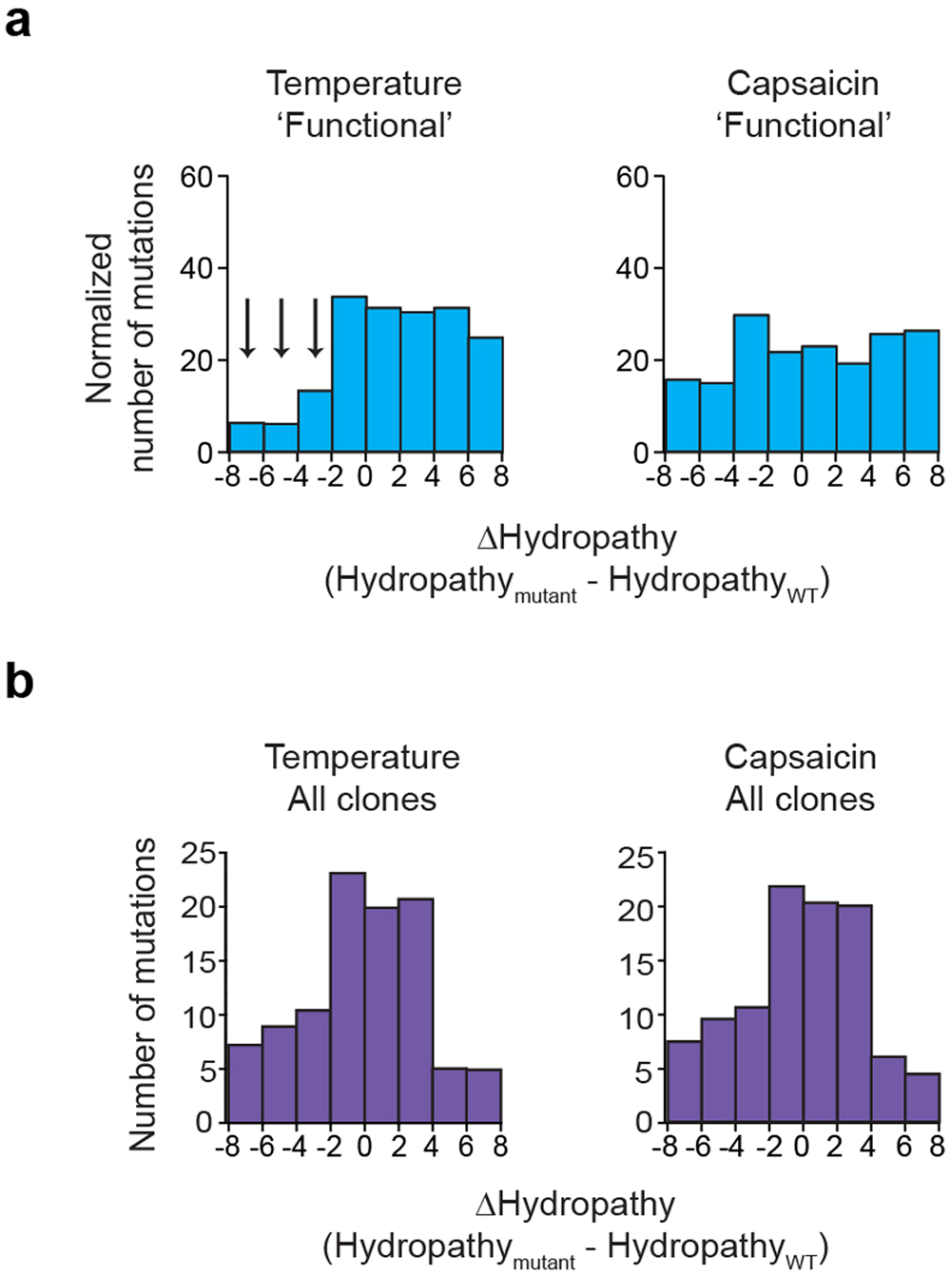
Hydropathy changes induced by mutations. (**a**) Histograms showing the change in hydropathy (hydropathy_mutant_ − hydropathy_WT_) for mutations in ‘functional’ clones screened with temperature or capsaicin. Data is normalized so that the sum of a bin from the ‘functional’ group and the respective bin from the ‘less functional’ group is equal to 100%. (**b**) Change in hydropathy for all 1,110 temperature- and 1,145 capsaicin-characterized mutations.

As introduced before, a proposed mechanism suggests that heat capacity mediated by exposure of hydrophobic residues to solvent may provide high temperature sensitivity of TRP channels^40^. We hypothesized that mutations that decrease the hydrophobicity of amino acids will reduce overall temperature sensitivity, whereas mutations that maintain or increase hydrophobicity will less likely affect it. To test our hypothesis, we analyzed how hydrophobicity was changed in our identified mutations. Specifically, we used a Kyte-Doolittle hydropathy scale to calculate the change in hydropathy (hydropathy_mutant_ − hydropathy_WT_)^43^. We then generated histograms of the number of mutations as a function of the changes in hydrophobicity (Fig. 4a). As we expected from our prediction, the analysis showed that mutations that cause a large decrease in hydropathy (∆hydropathy < −2) are not frequently found (9.1% or 26 out of 287 mutations) within the temperature ‘functional’ mutations, whereas mutations without changes or with moderate and strong increases in hydropathy (∆hydropathy > −2) occur frequently (90.9% or 261 out of 287 mutations). On the contrary, in the capsaicin ‘functional’ library mutations with large decrease in hydropathy (∆hydropathy < −2) are more frequent (25.8% or 64 out of 248 mutations) and mutations without changes or with moderate and strong increases in hydropathy (∆hydropathy > −2) are less frequent (74.2% or 184 out of 248 mutations). To statistically test this observation for both, temperature- and capsaicin-characterized mutations, we performed a likelihood ratio test and found that the number of temperature- and capsaicin-characterized mutations with Δhydropathy < −2 is significantly different (p value = 4.6 × 10^−129^) from the number of mutations with a Δhydropathy > −2 (see Methods). This indicates that mutations in TRPV1 that strongly reduce hydrophobicity may cause a decrease in overall temperature response possibly by affecting specific parts of the activation process, such as temperature sensing or gating. This result shows that the temperature activation mechanism may be more sensitive to mutations that decrease hydrophobicity as compared to the capsaicin activation mechanism. Importantly, this pronounced statistical difference in stimulus specificity is not caused by a bias of the mutant library and clone selection, as histograms representing hydrophobicity changes of all 2,255 mutations are nearly identical for both temperature and capsaicin (Fig. 4b). Taken together, these results correlate strong decreases in hydrophobicity of certain amino acids as being underrepresented in clones normal for temperature activation, but not in clones normal for capsaicin activation and provide a potential mechanistic link between temperature activation of TRPV1 to changes in hydrophobicity.

**Figure 5:**
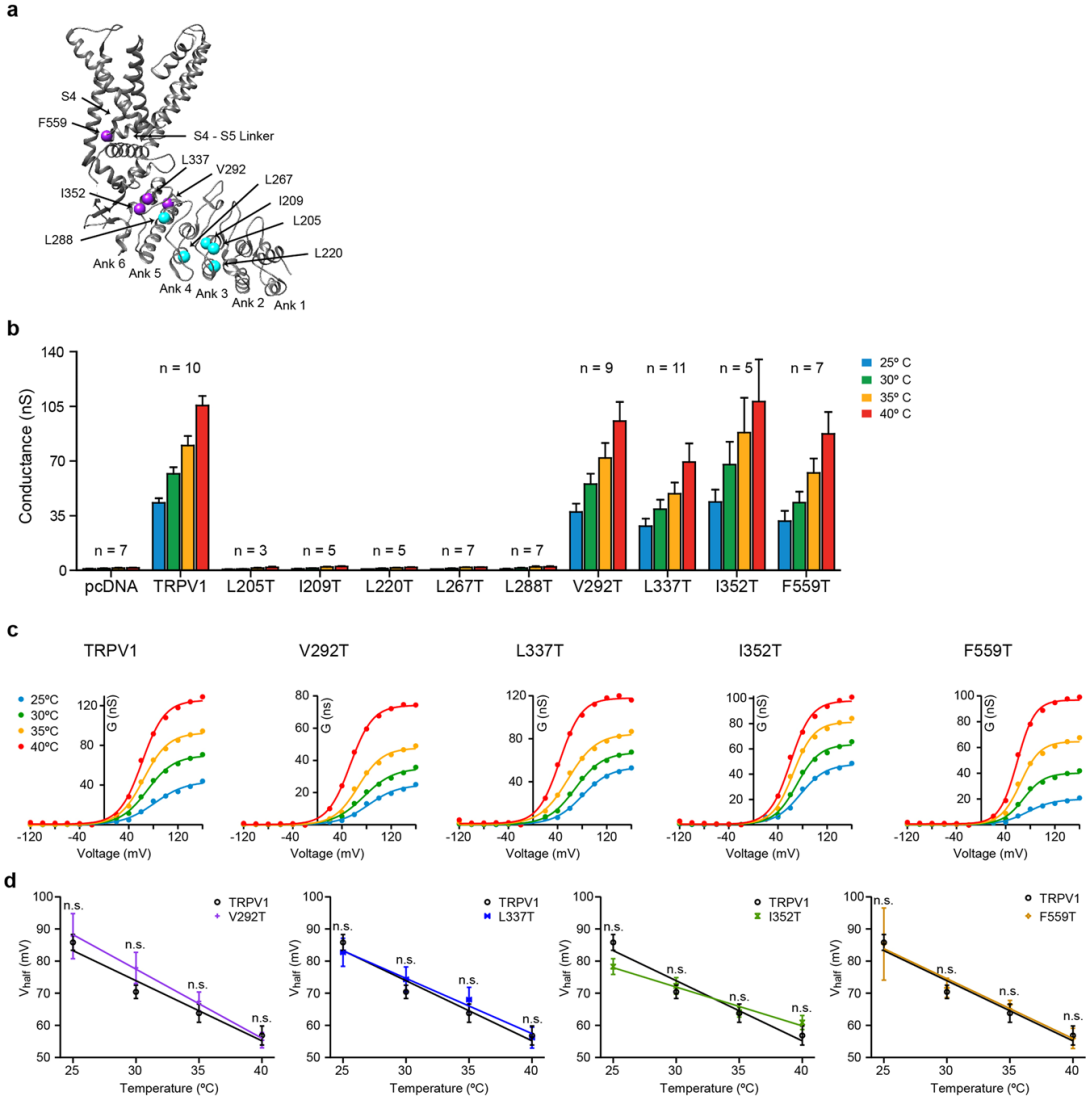
Heat sensitivity of TRPV1 single-point mutants with mutations that neutralized amino acid hydrophobicity. (**a**) Structural location of all the residues mutated in the ankyrin repeats and the beginning of the S4-S5 linker. In cyan are residues that when mutated to Thr, lead to non-functional channels. In purple are residues that, when mutated to Thr, do not affect temperature activation. (**b**) Average conductance at +160 mV as a function of temperature for mutants. Data represent mean ± SEM. N on top of the bars represent number of individual patches. (**c**) Conductance-voltage (G-V) relationship of plateau current for wild-type TRPV1, V292T, L337T, I352T, and F559T at different temperatures. (**d**) Voltage of half maximal activation (V_half_) for wild-type TRPV1, V292T, L337T, I352T, and F559T as a function of temperature. V_half_ was obtained from the fitting parameters of a Boltzmann distribution to the G-V. Data represent mean ± SEM. Lines are linear fits to the data. Wild-type TRPV1 n = 7, V292T n = 9, L337T n = 11, I352T n = 5, and F559T n = 7. Each V_half_ value was tested for statistical significance using an unpaired Student’s t-test. n.s. indicates not significant (p > 0.05).

Our results show on the ensemble level that hydrophobicity of amino acid is correlated with normal temperature activation of TRPV1. To test this mechanism further we engineered single-point mutations that neutralized amino acid hydrophobicity (Thr) for eight positions within the ankyrin repeats that are highly conserved between individual repeats of rat TRPV1, and one aligning position in the S4 that had been found to confer temperature sensitivity to K_v_1.2 (see Methods) (Fig. 5a)^29, 41^. We transiently expressed all constructs in HEK293 cells and measured currents upon voltage step protocols (−120 to +160 mV) at different temperatures (25 °C, 30 °C, 35 °C, and 40 °C) using patch-clamp electrophysiology (Fig. 5). We probed temperature sensitivity by measuring the voltage of half maximal activation (V_half_) as a function of temperature and tested how titrating amino acid hydrophobicity of residues affects overall temperature sensitivity of TRPV1^3, 44^. While mutations at five positions within the ankyrin repeats (L205T, I209T, L220T, L267T, and L288T) lead to non-functional channels, mutations V292T, L337T, I352T and, F559T resulted in channels that had conductance and V_half_ values with temperature dependences indistinguishable from wild-type TRPV1, suggesting that hydrophobicity at these specific residues does not contribute to temperature activation (Fig. 5b–d).

## Discussion

We set out to find the structures and mechanism by which temperature activates TRPV1 ion channels. We used a combination of random mutagenesis, high-throughput functional screening and deep sequencing to identify and correlate the effect of 535 mutations on channel activation by temperature or capsaicin. Deep mutational scanning is becoming a powerful method for the unbiased probing of protein function, largely due to rapid advances in sequencing technology. The unbiased and high-throughput nature of deep mutational scanning seems particularly suited for addressing the problem of temperature activation because it has thus far escaped approaches that are more targeted. While this approach had previously been used for the study of soluble proteins, to our knowledge this is the first case in which it has been used to study an ion channel^45–47^.

However, deep mutational scanning, and our approach in particular, has two technical caveats which must be considered. First, for an ideal investigation of structure-function relationships, each amino acid would be mutated into every other of the 19 possible amino acids or even be replaced by unnatural amino acids. However, due to the degeneration of the genetic code, only a subset of coding mutations is attainable by error-prone PCR. Our library covered approximately 14% of this sequence space (see Methods). Clearly, the library was not mutated to saturation and thus, many residues critical for temperature or capsaicin activation were likely missed. Nevertheless, we have identified 535 mutations, which is vastly surpassing the number of TRPV1 mutations characterized thus far^42^. Second, we functionally screened clones using a calcium-based fluorescence assay that measures ion channel activity only indirectly. Ideally, ion channels should be functionally characterized with electrophysiological measurements to precisely determine channel open probability, but the method lacks the throughput required for screening thousands of mutants efficiently. Still, calcium based fluorescence assays have been used many times to reliably measure activation of TRP channels using a variety of agonists^2, 20, 48^. Moreover, since we focused only on functional clones, mutations that cause a loss of function perhaps due to a decrease in channel expression, protein misfolding, changes in ion permeation, overlap of multiple mutations in one clone, or through other unspecific mechanisms are not compromising the analysis we performed. For example, multiple different mutations would be classified as ‘less functional’ if they originated from the same clone containing only a single mutation that renders it non-functional.

Our analysis of the temperature ‘functional’ and capsaicin ‘functional’ mutations shows that a large decrease in amino acid hydrophobicity negatively correlates with the likelihood of ion channel activation by temperature. This result is specific for temperature activation, as capsaicin activation is not drastically affected by amino acid hydrophobicity. Importantly, this statistical observation matches a key prediction of the proposed heat capacity mechanism, and thus provides the first indication that this mechanism may underlie temperature activation in TRPV1. More evidence comes from our analysis of the location of mutations. Mapping of all temperature ‘functional’ mutations onto the primary structure shows that all structurally distinct domains, such as ankyrin repeats and transmembrane domains are, to a large extent, tolerant to mutations. This result argues for the absence of a distinct and coherent ‘temperature sensor domain’, in which all residues contribute to temperature sensitivity and might explain why previous studies identified multiple domains or several scattered residues within a single domain^18, 20–22, 28–32, 34, 35, 49^. However, it is theoretically possible that the mutations we introduced and identified are not sufficiently disruptive for temperature activation and that an existing ‘temperature sensor domain’ has not emerged from our screen. The fact that the library is not sampling the majority of the sequence space is one possible explanation of why a putative temperature domain did not emerge. It is also important to point out that mutations might affect temperature gating instead of sensing, both of which are still unknown mechanisms.

To further test our statistical observation we introduced eight single-point mutations that neutralized the hydrophobicity of residues in the ankyrin repeats and one single-point mutation in an aligning position in the S4 that was found to make K_v_1.2 temperature sensitive. However, five of the introduced mutations in the ankyrin repeats abolished channel activation. On the other hand, the other three ankyrin repeat nor the S4 mutations were sufficient to affect temperature activation of the channel. This result indicates that not all hydrophobic residues contribute to temperature activation and supports our high-throughput results that a subset and sparse population of residues contribute to temperature activation. In fact, Clapham and Miller estimated that ~20 side chains per subunit might be required to generate a substantial change in heat capacity^40^. However, the intuitive assumption that the effects of all hydrophobic residues are independent and thus additive might not be the case in the context of a protein structure. For example, temperature might drive a conformational change that simultaneously exposes several amino acids, which are structurally in close proximity to the solvent. Conversely, mutating only one of these amino acids might inhibit this conformational change altogether, thus eliminating the contribution of all locally involved amino acids. Due to this complex interdependence we do not expect all single amino acids contributing equally and therefore single-point mutations paired with functional analysis might only be able to identify amino acids that contribute most prominently. Moreover, mutations reducing hydrophobicity might additionally affect other activation mechanisms so that effects cannot be assigned unambiguously to a temperature mechanism.

Clearly, investigating the relationship between amino acid hydrophobicity and temperature sensitivity for all amino acids in TRPV1 is an enormous task. Deep mutational scanning with better library saturation and more accurate functional characterization seems specifically tailored to solve it. The correlative relationship demonstrated here is a first indication of the collective importance of hydrophobicity for temperature activation and the distributed nature of temperature sensitive structures. Additional evidence from protein calorimetry, cysteine accessibility, and high-resolution structures at different temperatures is needed to fully probe and perhaps establish a heat capacity mechanism of temperature activation.

## Methods

### Library Generation and Functional Screening

Generation and screening of the TRPV1 random mutant library was described previously in detail^34^. Briefly, error-prone PCR was performed with the full-length rat *Trpv1* gene (Diversify PCR random mutagenesis kit, Clontech) and yielded an average mutation frequency of 2 amino acid mutations per clone. Mutated DNA was mini-prepped, normalized to 40 ng/μl and plated in quadruplets in 384-well clear bottom assay plates (Greiner), transfection reagent (Fugene 6.0, Roche), and HEK293 cells were added. Each 384-well plate contained separate wells with wild-type TRPV1 and pcDNA5 transfected cells as positive and negative controls, respectively. Two days after transfection cells were washed, loaded with the calcium sensitive fluorophore Fluo-3 and washed again. Plates were then transferred to a FLIPR-TETRA (Molecular Devices) plate reader to monitor the Fluo-3 fluorescence changes. The library was screened independently upon stimulation by heat (25 °C to 45 °C) and capsaicin (100 nM, final concentration).

### Categorization of Mutant Library

Maximal fluorescence responses (*x*) were calculated for each well after baseline subtraction. For each plate mean maximal responses ($\bar{x}$) and standard deviations (σ) were calculated for wild-type TRPV1 (${\bar{x}}_{TRPV1}\,{\rm{and}}\,{\sigma }_{TRPV1}$) and pcDNA5 (${\bar{x}}_{pcDNA}\,{\rm{and}}\,{\sigma }_{pcDNA}$) transfected cells. Plates where ${\bar{x}}_{TRPV1}$ and ${\bar{x}}_{pcDNA}$ was not consistent for three of the four wells were discarded. Clones were selected as ‘functional’ if ${x}_{clone} > {\bar{x}}_{TRPV1}-1{\sigma }_{TRPV1}$. Clones were classified as ‘less functional’ if ${x}_{clone} < {\bar{x}}_{TRPV1}-1{\sigma }_{TRPV1}$. Clones that did not consistently fulfill these criteria for at least three of the four wells were not considered. In total ~7,300 out of ~8,500 clones were selected and their cDNA was picked for sequence analysis.

### Sample Preparation and Sequencing

One µl cDNA of each clone was collected by hand and subsequently cDNA was pooled for each of the categories. Next, clones were digested with HindIII and Not1 restriction enzymes and the mutated TRPV1 insert was separated from pcDNA vector using gel electrophoresis. Mutated TRPV1 inserts were then gel-purified using a QIAquick^®^ gel extraction kit (QIAGEN) and concentrated to 40 ng/µl using a DNA concentrator kit (Zymo Research). Mutated inserts were then fragmented by sonication (Covaris), multiplexed and sequenced with 150 paired-end reads using the illumina HiSeq 2500 sequencer in rapid mode (Duke University Genome Sequencing and Analysis Core). All clones were sequenced together in a single lane. Each clone was sequenced with a coverage of more than 700,000x. All sequences had a Phred score of at least 34 (99.96% accuracy).

### Aligning and Mapping of Variants

Variants were aligned and mapped by the Duke University Genome Sequencing and Analysis Core. Adaptor sequences and low quality bases were removed using cutadapt^50^. Forward and reverse paired reads were then repaired using a custom script (Duke University Genome Sequencing and Analysis Core). Sequences were aligned to wild-type rat *Trpv1* using bwa for paired-end reads^51^. Variant calls were made using SNVer^52^.

### Determination of Variant Call Accuracy and Mutational Frequency

We postulated that sequences from clones categorized as ‘functional’ should not contain any stop codons before the pore domain (Q560) and applied a binomial distribution with 95% confidence level to empirically determine the variant probability to 0.0016. All mutations with a confidence level below 95% were discarded. The same p-value and 95% confidence interval was then applied to all mutations. The frequency of mutation for each base pair was calculated by dividing the total number of reads of a given residue by the number of times a non-silent substitution was read. The coverage at base pair level was calculated using the formula:

1 $$(\frac{\#\,of\,identified\,base\,pair\,changes}{(\#\,of\,bases\,in\,WT\,TRPV1)(\#\,of\,possible\,base\,changes)})=(\frac{5546}{(2514)(3)})=73 \% $$

The library coverage on an amino acid level was calculated using the formula:

2 $$(\frac{\#\,of\,identified\,amino\,acid\,changes}{(\#of\,amino\,acids\,in\,WT\,TRPV1)(\#\,of\,possible\,amino\,acid\,changes)})=(\frac{2255}{(838)(19)})=14 \% $$

### Alignment of TRPV1 ankyrin repeats and TRPV1 S4

Alignments were performed on Clustal Omega^53, 54^. For aligning of ankyrin repeats the start and end of each repeat was identified using the apo high-resolution structure^29^.

### High-Resolution Structure Illustrations

Structure illustrations were made using the unliganded (apo) TRPV1 high-resolution structure (Liao *et al*., 2013; PDB ID: 3J5P) with the UCSF Chimera program^55^.

### Site-directed Mutagenesis

All mutations were introduced into a wild-type rat TRPV1 ligated to a pcDNA 3.1 expression vector. Mutants were generated using a QuickChange Site-Directed Mutagenesis Kit (Agilent). All mutants were fully sequenced using Sanger sequencing.

### Cell Culture

HEK293t cells were cultured in Dulbecco’s Modified Eagle’s Medium (DMEM) (Life Technologies) supplemented with 10% fetal bovine serum (Clontech Laboratories), 50 unit/mL penicillin (Life Technologies), and 50 mg/mL streptomycin (Life Technologies). Cells were transiently transfected in 6-well plates in the presence of 10 µM ruthenium red using Fugene 6.0 (Promega) ~48 hr before recording. All cells were co-transfected with GFP or YFP as a fluorescent reporter. Transfected cells were re-plated at low density at least 16 hr before recording in 10 mm glass coverslip (Warner Instruments) coated with Poly-L-lysine (Sigma) and laminin (Sigma).

### Electrophysiology

Patch-clamp recordings were performed using an EPC10 amplifier and Patchmaster software (HEKA Elektronik) in the whole cell configuration. Data were sampled at 5 kHz and filtered at 2.9 kHz. Borosilicate glass pipettes (Sutter Instruments) had a resistance of 3–5 MΩ when filled with pipette buffer solution (in mM): 150 NaCl, 3 MgCl_2_, 5 EGTA, 10 HEPES, pH = 7.2 with NaOH. The bath solution was (in mM): 150 NaCl, 6 CsCl, 1.5 CaCl_2_, 1 MgCl_2_, 10 glucose, 10 HEPES, pH = 7.4 with NaOH. Solutions were applied through a gravity-driven perfusion, and temperature was controlled using a CL-100 temperature controller (Warner Instruments) and a SC-20 dual in-line heater (Harvard Apparatus). Temperature was measured using a TA-29 thermistor (Warner Instruments) placed near the recorded cell. Heat activation experiments were done by increasing the temperature from 25 °C to 40 °C in 5 °C intervals. At every temperature interval (25 °C, 30 °C, 35 °C, and 40 °C) we measured channel activation with a voltage step from −120 mV to +160 mV at 20 mV increases for 200 ms and used these data to calculate conductance-voltage (G-V) relationships. The currents for the G-V curves were obtained from plateau currents at the last 50 ms of the voltage step. G-V curves were then fitted with Boltzmann functions on Igor Pro (WaveMetrics) and the V_half_ values were obtained from the fitting parameters. Recordings were only analyzed for patches with an initial seal resistance of at least 1 GΩ, voltage-induced current at +160 mV and 25 °C of at least 500 pA and series resistance (R_series_) < 10 MΩ at 25 °C for wild-type TRPV1 and mutant channels.

### Data Analysis and Statistical Tests

Mutation selection was performed with Excel (Microsoft). Graphs were made using Prism (GraphPad Software, Inc.) and Igor Pro (WaveMetrics). Illustrations were made in Illustrator (Adobe Systems). Histograms of gap lengths between mutations were generated by counting gap lengths (for temperature ‘functional’ or capsaicin ‘functional’ mutations), or by generating 10 random distributions of 287 (temperature) or 248 (capsaicin) mutations over 838 positions with Excel (Microsoft) and then counting gap lengths. Electrophysiological data were analyzed with Igor Pro (WaveMetrics).

Statistical analysis of hydropathy histograms was performed on R using a custom made script. We used a likelihood ratio (LRT) to test whether the frequency of temperature-characterized mutations with ∆hydropathy > −2 is the same between each of the two functional categories. V_half_ statistical analysis was done using an unpaired Student’s t-test by comparing wild-type TRPV1 to each mutant.

## Electronic supplementary material

Supplementary Information and FigureS1

## References

[1] Nilius, B. & Flockerzi, V. *Mammalian Transient Receptor Potential* (*TRP*) *Cation Channels*. **I**, (Springer, 2014). 25296415

[2] Caterina MJ (1997). The capsaicin receptor: a heat-activated ion channel in the pain pathway. Nature.

[3] Voets T (2004). The principle of temperature-dependent gating in cold- and heat-sensitive TRP channels. Nature.

[4] Dhaka A (2009). TRPV1 is activated by both acidic and basic pH. J. Neurosci..

[5] Siemens J (2006). Spider toxins activate the capsaicin receptor to produce inflammatory pain. Nature.

[6] Bohlen CJ (2010). A bivalent tarantula toxin activates the capsaicin receptor, TRPV1, by targeting the outer pore domain. Cell.

[7] Caterina MJ (2000). Impaired nociception and pain sensation in mice lacking the capsaicin receptor. Science (80-.).

[8] Davis JB (2000). Vanilloid receptor-1 is essential for inflammatory thermal hyperalgesia. Nature.

[9] Jordt S, Julius D, Francisco S (2002). Molecular Basis for Species-Specific Sensitivity to ‘Hot’ Chili Peppers. Cell.

[10] Yang F (2015). Structural mechanism underlying capsaicin binding and activation of the TRPV1 ion channel. Nat. Chem. Biol..

[11] Jordt S-E, Tominaga M, Julius D (2000). Acid potentiation of the capsaicin receptor determined by a key extracellular site. Proc. Natl. Acad. Sci. USA.

[12] Ryu S, Liu B, Yao J, Fu Q, Qin F (2007). Uncoupling proton activation of vanilloid receptor TRPV1. J. Neurosci..

[13] Hu H, Grandl J, Bandell M, Petrus M, Patapoutian A (2009). Two amino acid residues determine 2-APB sensitivity of the ion channels TRPV3 and TRPV4. Proc. Natl. Acad. Sci. USA.

[14] Bandell M (2006). High-throughput random mutagenesis screen reveals TRPM8 residues specifically required for activation by menthol. Nat. Neurosci..

[15] Aggarwal SK, MacKinnon R (1996). Contribution of the S4 segment to gating charge in the Shaker K+ channel. Neuron.

[16] Seoh S, Sigg D, Papazian DM, Bezanilla F (1996). Voltage-Sensing Residues in the S2 and S4 Segments of the Shaker K+ Channel. Neuron.

[17] Cao E, Cordero-Morales JF, Liu B, Qin F, Julius D (2013). TRPV1 channels are intrinsically heat sensitive and negatively regulated by phosphoinositide lipids. Neuron.

[18] Moparthi L (2014). Human TRPA1 is intrinsically cold- and chemosensitive with and without its N-terminal ankyrin repeat domain. Proc. Natl. Acad. Sci. USA.

[19] Yao J, Liu B, Qin F (2010). Kinetic and energetic analysis of thermally activated TRPV1 channels. Biophys. J..

[20] Jabba S (2014). Directionality of temperature activation in mouse TRPA1 ion channel can be inverted by single-point mutations in ankyrin repeat six. Neuron.

[21] Yao J, Liu B, Qin F (2011). Modular thermal sensors in temperature-gated transient receptor potential (TRP) channels. Proc. Natl. Acad. Sci. USA.

[22] Grandl J (2008). Pore region of TRPV3 ion channel is specifically required for heat activation. Nat. Neurosci..

[23] Gao Y, Cao E, Julius D, Cheng Y (2016). TRPV1 structures in nanodiscs reveal mechanisms of ligand and lipid action. Nature.

[24] Doyle DA (2013). The Structure of the Potassium Channel: Molecular Basis of K+ Conduction and Selectivity. Science (80-.).

[25] Latorre R, Brauchi S, Orta G, Zaelzer C, Vargas G (2007). ThermoTRP channels as modular proteins with allosteric gating. Cell Calcium.

[26] White SH, Wimley WC (1999). Membrane protein folding and stability: Physical principles. Ann. Rev. Biophys. Biomol. Struct.

[27] Haltia T, Freire E (1995). Forces and factors that contribute to the structural stability of membrane proteins. Biochim. Biophys. Acta.

[28] Yao, J., Liu, B. & Qin, F. Pore turret of thermal TRP channels is not essential for temperature sensing. *Proc. Natl. Acad. Sci. USA* **107**, E125; author reply E126–7 (2010). 10.1073/pnas.1008272107 PMC2922611 20660307

[29] Liao M, Cao E, Julius D, Cheng Y (2013). Structure of the TRPV1 ion channel determined by electron cryo-microscopy. Nature.

[30] Cordero-Morales JF, Gracheva EO, Julius D (2011). Cytoplasmic ankyrin repeats of transient receptor potential A1 (TRPA1) dictate sensitivity to thermal and chemical stimuli. Proc. Natl. Acad. Sci. USA.

[31] Kang K (2012). Modulation of TRPA1 thermal sensitivity enables sensory discrimination in Drosophila. Nature.

[32] Zhong L, Bellemer A, Yan H, Honjo K, Robertson J (2012). Thermosensory and non-thermosensory isoforms of Drosophila melanogaster TRPA1 reveal heat sensor domains of a thermoTRP channel. Cell Rep.

[33] Gracheva EO (2010). Molecular basis of infrared detection by snakes. Nature.

[34] Grandl J (2010). Temperature-induced opening of TRPV1 ion channel is stabilized by the pore domain. Nat. Neurosci..

[35] Chen J (2013). Species differences and molecular determinant of TRPA1 cold sensitivity. Nat. Commun..

[36] Cao E, Liao M, Cheng Y, Julius D (2013). TRPV1 structures in distinct conformations reveal activation mechanisms. Nature.

[37] Paulsen CE, Armache J-P, Gao Y, Cheng Y, Julius D (2015). Structure of the TRPA1 ion channel suggests regulatory mechanisms. Nature.

[38] Zubcevic L (2016). Cryo-electron microscopy structure of the TRPV2 ion channel. Nat. Struct. Mol. Biol..

[39] Huynh KW (2016). Structure of the full-length TRPV2 channel by cryo-EM. Nat. Commun..

[40] Clapham DE, Miller C (2011). A thermodynamic framework for understanding temperature sensing by transient receptor potential (TRP) channels. Proc. Natl. Acad. Sci. USA.

[41] Chowdhury S, Jarecki BW, Chanda B (2014). A molecular framework for temperature-dependent gating of ion channels. Cell.

[42] Winter Z (2013). Functionally important amino acid residues in the transient receptor potential vanilloid 1 (TRPV1) ion channel–an overview of the current mutational data. Mol. Pain.

[43] Kyte J, Doolittle RF (1982). A Simple Method for Displaying the Hydropathic Character of a Protein. J. Mol. Biol..

[44] Voets T (2012). Quantifying and Modeling the Temperature-Dependent Gating of TRP Channels. Rev. Physiol. Biochem. Pharmacol.

[45] Adkar BV (2012). Protein model discrimination using mutational sensitivity derived from deep sequencing. Structure.

[46] Holmqvist E, Reimegård J, Wagner EGH (2013). Massive functional mapping of a 5′-UTR by saturation mutagenesis, phenotypic sorting and deep sequencing. Nucleic Acids Res.

[47] Fowler DM (2010). High-resolution mapping of protein sequence-function relationships. Nat. Methods.

[48] McKemy D, Neuhausser W, Julius D (2002). Identification of a cold receptor reveals a general role for TRP channels in thermosensation. Nature.

[49] Yang F, Cui Y, Wang K, Zheng J (2010). Thermosensitive TRP channel pore turret is part of the temperature activation pathway. Proc. Natl. Acad. Sci. USA.

[50] Martin M (2011). Cutadapt removes adapter sequences from high-throughput sequencing reads. Eur. Mol. Biol. Netw.

[51] Li H, Durbin R (2009). Fast and accurate short read alignment with Burrows-Wheeler transform. Bioinformatics.

[52] Wei Z, Wang W, Hu P, Lyon GJ, Hakonarson H (2011). SNVer: a statistical tool for variant calling in analysis of pooled or individual next-generation sequencing data. Nucleic Acids Res.

[53] Sievers F (2011). Fast, scalable generation of high-quality protein multiple sequence alignments using Clustal Omega. Mol. Syst. Biol..

[54] Goujon M (2010). A new bioinformatics analysis tools framework at EMBL-EBI. Nucleic Acids Res.

[55] Pettersen EF (2004). UCSF Chimera - A visualization system for exploratory research and analysis. J. Comput. Chem..

