# Supplementary material for: TRPV1 temperature activation is specifically sensitive to strong decreases in amino acid hydrophobicity
Source: Sci Rep. 2017 Apr 3;7:549. doi: 10.1038/s41598-017-00636-4 (PMC5428820; doi:10.1038/s41598-017-00636-4)
Supplement: Supplementary file 1 — Supplementary Information and FigureS1 [file 41598_2017_636_MOESM1_ESM.pdf]

TRPV1 temperature activation is specifically sensitive to strong decreases in amino acid hydrophobicity

Jason O. Sosa-Pagán[1], Edwin S. Iversen[2], and Jörg Grandl[1,*]

[1]Department of Neurobiology, Duke University Medical Center, Durham, NC 27710, USA.

[2]Department of Statistical Science, Duke University, Durham, NC 27710, USA.

[*]Correspondence: Jörg Grandl, Duke University Medical Center, Box 103030 Med Ctr, Durham, NC 27710, USA, grandl@neuro.duke.edu, phone 919-684-1144

Supplementary Table S1. List of temperature- and capsaicin-characterized 'functional' mutations with their corresponding change in hydropathy
Identified coding mutations listed for each functional category (wild-type amino acid/position/mutated amino acid).

Temperature-characterized mutations		Capsaicin-characterized mutations	
'Functional clones'	ΔHydropathy (Hydropathy$_{mutant}$ – Hydropathy$_{WT}$)	'Functional clones'	ΔHydropathy (Hydropathy$_{mutant}$ – Hydropathy$_{WT}$)
M1I	2.6	R4W	3.6
R4L	8.3	R4L	8.3
D8Y	2.2	S6N	-2.7
E11G	3.1	E11V	7.7
P15T	0.9	C21R	-7.0
P15Q	-1.9	P29H	-1.6
Q17K	-0.4	P35T	0.9
E18G	3.1	V36D	-7.7
E18D	0.0	K37E	0.4
C21Y	-3.8	K37M	5.8
D23Y	2.2	R46H	1.3
P24H	-1.6	R46C	7.0
D26V	7.7	S55L	4.6
D28Y	2.2	L61M	-1.9
P29H	-1.6	L61Q	-7.3
P35T	0.9	P64H	-1.6
V36D	-7.7	E67D	0.0
K37E	0.4	G69W	-0.5
H39N	-0.3	L70P	-5.4
H39L	7.0	P74H	-1.6
F41L	1.0	I75N	-8.0
R44M	6.4	V81A	-2.4
R46S	3.7	L82P	-5.4
R48W	3.6	G88W	-0.5
G51W	-0.5	G88V	4.6
K52E	0.4	D89V	7.7
K52N	0.4	S93R	-3.7
D54Y	2.2	S97L	4.6
S55P	-0.8	Q99K	-0.4
P60T	0.9	Q99R	-1.0
L61M	-1.9	S101T	0.1
P64H	-1.6	E106V	7.7
G69W	-0.5	P109Q	-1.9
P74H	-1.6	L111P	-5.4
I76N	-8.0	S116C	3.3
L82I	0.7	D119E	0.0

T83A	2.5	R138W	3.6
P87L	5.4	G168W	-0.5
G88W	-0.5	Q169R	-1.0
G88V	4.6	R181L	8.3
D89V	7.7	S185C	3.3
S93C	3.3	S193C	3.3
Q99K	-0.4	D196V	7.7
Q99R	-1.0	S197C	3.3
D100G	3.1	K200N	0.4
P109Q	-1.9	N213S	2.7
R114L	8.3	Q228H	0.3
R115G	4.1	A230V	2.4
R115S	3.7	A231T	-2.5
D119E	0.0	G233R	-4.1
Q123H	0.3	G233E	-3.1
Q127L	7.3	G233W	-0.5
E130G	3.1	G233V	4.6
R138S	3.7	K238E	0.4
K141E	0.4	K240N	0.4
D145Y	2.2	G241W	-0.5
F148L	1.0	L261Q	-7.3
D150Y	2.2	V264A	-2.4
E152D	0.0	K265M	5.8
K155N	0.4	A275T	-2.5
M162V	2.3	I277V	-0.3
G168W	-0.5	R280L	8.3
G168V	4.6	K303E	0.4
Q169R	-1.0	F304L	1.0
Q169H	0.3	T306I	5.2
D171Y	2.2	G316V	4.6
L175M	-1.9	H320P	1.6
R181L	8.3	T329S	-0.1
K182N	0.4	S343N	-2.7
D184Y	2.2	G344W	-0.5
S193C	3.3	G347W	-0.5
D196Y	2.2	R355M	6.4
D196V	7.7	V377M	-2.3
S197C	3.3	L381I	0.7
Y199N	-2.2	I387V	-0.3
Q202H	0.3	T389S	-0.1
R212L	8.3	C390R	-7.0
N213S	2.7	S402G	0.4
M214I	2.6	P407T	0.9
L219I	0.7	P407H	-1.6
G224V	4.6	R409S	3.7

A230V	2.4	D411N	0.0
G233W	-0.5	D411V	7.7
G233V	4.6	N419D	0.0
D234Y	2.2	W426L	4.7
K238E	0.4	F429L	1.0
T239A	2.5	R432H	1.3
T239S	-0.1	I433V	-0.3
G241W	-0.5	F436I	1.7
L252M	-1.9	F439L	1.0
L254M	-1.9	I446V	-0.3
V264A	-2.4	I447T	-5.2
F266L	1.0	Y453H	-1.9
Q269H	0.3	E458G	3.1
S271Y	-0.5	L460M	-1.9
R280L	8.3	P462T	0.9
D281Y	2.2	P462H	-1.6
L291M	-1.9	K464R	-0.6
D300Y	2.2	L465P	-5.4
K303E	0.4	N467S	2.7
F304L	1.0	V469A	-2.4
G316V	4.6	D471G	3.1
H320R	-1.3	Y472N	-2.2
P321H	-1.6	Y472C	3.8
T322S	-0.1	T476S	-0.1
E327G	3.1	I479T	-5.2
I328N	-8.0	S483L	4.6
K332R	-0.6	V486A	-2.4
S343N	-2.7	F488L	1.0
G344W	-0.5	F488S	-3.6
G347W	-0.5	F489S	-3.6
G347V	4.6	F490L	1.0
Q354K	-0.4	F490S	-3.6
R355M	6.4	F496S	-3.6
R363L	8.3	Q498R	-1.0
F369L	1.0	Q498L	7.3
G375W	-0.5	S502T	0.1
V377M	-2.3	L503I	0.7
S379Y	-0.5	K504M	5.8
L381I	0.7	S505G	0.4
D383Y	2.2	F507I	1.7
D388Y	2.2	F522L	1.0
T389S	-0.1	M523T	-2.6
C390R	-7.0	M523V	2.3
K392N	0.4	M523L	1.9
L396M	-1.9	F531L	1.0

P407T	0.9	S532G	0.4
P407H	-1.6	R534L	8.3
R409S	3.7	M541T	-2.6
H410N	-0.3	M541V	2.3
H410Q	-0.3	V542A	-2.4
D411Y	2.2	L545M	-1.9
L418F	-1.0	M547L	1.9
N419D	0.0	M552T	-2.6
R420L	8.3	M552V	2.3
W426L	4.7	Y555F	4.1
W426C	3.4	T556A	2.5
R432H	1.3	I564T	-5.2
F438L	1.0	K571R	-0.6
F439A	-1.0	M572T	-2.6
M445L	1.9	M572V	2.3
I446V	-0.3	C578Y	-3.8
I447T	-5.2	F582L	1.0
L460M	-1.9	F582S	-3.6
P461H	-1.6	L585I	0.7
P462T	0.9	T593A	2.5
P462H	-1.6	T593S	-0.1
K464R	-0.6	V596A	-2.4
N467S	2.7	G602W	-0.5
V469A	-2.4	N604S	2.7
G470W	-0.5	L607P	-5.4
G470V	4.6	M609K	-5.8
Y472C	3.8	M609V	2.3
I479T	-5.2	E610G	3.1
S481Y	-0.5	H614L	7.0
V486A	-2.4	R617W	3.6
F488L	1.0	R617L	8.3
F490L	1.0	S619T	0.1
G492W	-0.5	C621R	-7.0
Q494K	-0.4	K622R	-0.6
Q498R	-1.0	K622M	5.8
R500L	8.3	P623L	5.4
P501Q	-1.9	L630M	-1.9
L503I	0.7	N652D	0.0
F522L	1.0	I679V	-0.3
M523V	2.3	M682I	2.6
S526Y	-0.5	K688R	-0.6
F531L	1.0	Q700R	-1.0
Q533H	0.3	T704A	2.5
M541T	-2.6	T708A	2.5
M541V	2.3	T708I	5.2

F543L	1.0	K710R	-0.6
S544Y	-0.5	S711G	0.4
L545M	-1.9	K714R	-0.6
M547L	1.9	C715S	-3.3
M547I	2.6	C715R	-7.0
M552T	-2.6	M716K	-5.8
M562I	2.6	M716T	-2.6
R575G	4.1	M716R	-6.4
F582L	1.0	R717G	4.1
F582S	-3.6	R717M	6.4
L585I	0.7	K718E	0.4
F587L	1.0	K718R	-0.6
G602W	-0.5	A719V	2.4
K603R	-0.6	F720S	-3.6
S606Y	-0.5	K724E	0.4
L607M	-1.9	Q727R	-1.0
S611Y	-0.5	G729W	-0.5
R617W	3.6	F730Y	-4.1
R617L	8.3	F730L	1.0
G618W	-0.5	T731A	2.5
K622M	5.8	D733G	3.1
S626Y	-0.5	R739L	8.3
L630M	-1.9	W740R	-3.6
S632Y	-0.5	R743M	6.4
F638L	1.0	R743S	3.7
F640L	1.0	V744A	-2.4
M644I	2.6	E746K	-0.4
D646Y	2.2	E746G	3.1
F649L	1.0	W752R	-3.6
N652D	0.0	N753D	0.0
D654Y	2.2	T754P	-0.9
I660V	-0.3	N755D	0.0
L662M	-1.9	V756E	-7.7
L669I	0.7	V756A	-2.4
L673I	0.7	N765D	0.0
I679V	-0.3	N765S	2.7
L681I	0.7	C766R	-7.0
M682I	2.6	E767D	0.0
E684D	0.0	V769I	0.3
K688R	-0.6	V769D	-7.7
E692D	0.0	R771H	1.3
K694N	0.4	T772S	-0.1
Q700H	0.3	L773M	-1.9
T708A	2.5	L773Q	-7.3
S711G	0.4	L773P	-5.4

K714R	-0.6	F775S	-3.6
C715R	-7.0	S776Y	-0.5
M716K	-5.8	L777P	-5.4
M716T	-2.6	S783P	-0.8
M716I	2.6	G784W	-0.5
R717M	6.4	N786S	2.7
S722Y	-0.5	W787R	-3.6
G729W	-0.5	K788R	-0.6
F730Y	-4.1	N789S	2.7
F730L	1.0	F790L	1.0
T731A	2.5	L792M	-1.9
G734C	2.9	V793A	-2.4
K735N	0.4	R797M	6.4
D737Y	2.2	D798G	3.1
W740R	-3.6	D798V	7.7
R743M	6.4	A799V	2.4
R743S	3.7	R804S	3.7
V744A	-2.4	E811G	3.1
E746K	-0.4	V812F	-1.4
N748S	2.7	L814P	-5.4
W749L	4.7	K815E	0.4
W749C	3.4	H816Y	1.9
W752L	4.7	T818M	2.6
W752C	3.4	L821P	-5.4
N753S	2.7	E824G	3.1
N755D	0.0	D825G	3.1
I758T	-5.2	F829S	-3.6
D762Y	2.2	D831G	3.1
P763Q	-1.9	G836W	-0.5
N765D	0.0	G836V	4.6
E767V	7.7	E837G	3.1
E767D	0.0	K838N	0.4
L773M	-1.9		
F775S	-3.6		
S776Y	-0.5		
L777M	-1.9		
R781L	8.3		
S783P	-0.8		
G784W	-0.5		
G784V	4.6		
R785G	4.1		
R785I	9.0		
N786S	2.7		
K788R	-0.6		
K788N	0.4		

F790L	1.0		
L792M	-1.9		
L792P	-5.4		
V793A	-2.4		
L795I	0.7		
R797M	6.4		
R797S	3.7		
D798G	3.1		
D798V	7.7		
A799V	2.4		
R802L	8.3		
Q808K	-0.4		
E811G	3.1		
Q813K	-0.4		
T818M	2.6		
L821I	0.7		
E824G	3.1		
E824D	0.0		
E827V	7.7		
K830N	0.4		
D831G	3.1		
M833V	2.3		
M833I	2.6		
P835Q	-1.9		
G836W	-0.5		
G836V	4.6		

Supplementary Table S2. List of temperature- and capsaicin-characterized 'less functional' mutations with their corresponding change in hydropathy
Identified coding mutations listed for each functional category (wild-type amino acid/position/mutated amino acid).

Temperature-characterized mutations		Capsaicin-characterized mutations	
'Less functional clones'	ΔHydropathy ($Hydropathy_{mutant}$ – $Hydropathy_{WT}$)	'Less functional clones'	ΔHydropathy ($Hydropathy_{mutant}$ – $Hydropathy_{WT}$)
M1T	-2.6	M1I	2.6
A5V	2.4	Q3H	0.3
L7I	0.7	A5V	2.4
S9T	0.1	L7I	0.7
S9P	-0.8	D8Y	2.2
E10D	0.0	D8G	3.1
E10G	3.1	S9T	0.1
S12Y	-0.5	E10D	0.0
E13G	3.1	E11G	3.1
S14Y	-0.5	S12Y	-0.5
P16H	-1.6	S14Y	-0.5
S20Y	-0.5	S14T	0.1
P24T	0.9	P15T	0.9
P25Q	-1.9	P15Q	-1.9
D26Y	2.2	P16H	-1.6
R27I	9.0	Q17K	-0.4
P29T	0.9	E18G	3.1
C31Y	-3.8	E18D	0.0
P33Q	-1.9	S20Y	-0.5
P34H	-1.6	L22M	-1.9
P35Q	-1.9	L22Q	-7.3
P38H	-1.6	D23Y	2.2
I40N	-8.0	P24H	-1.6
I40V	-0.3	P24T	0.9
I40T	-5.2	P25T	0.9
R44S	3.7	D26G	3.1
R44W	3.6	D26Y	2.2
R46H	1.3	R27I	9.0
R48L	8.3	D28Y	2.2
G51V	4.6	P29T	0.9
G53C	2.9	N30I	8.0
D54V	7.7	K32M	5.8
E56D	0.0	P33Q	-1.9
S59Y	-0.5	P35Q	-1.9
P60H	-1.6	P38H	-1.6
D62Y	2.2	H39N	-0.3

C63Y	-3.8	H39R	-1.3
P64T	0.9	H39L	7.0
Y65N	-2.2	I40N	-8.0
E66D	0.0	I40T	-5.2
A71T	-2.5	I40V	-0.3
A71V	2.4	F41L	1.0
C73R	-7.0	T43I	5.2
P74T	0.9	R44W	3.6
I75T	-5.2	R44M	6.4
I76T	-5.2	R44S	3.7
I76F	-1.7	R46S	3.7
T77S	-0.1	R48L	8.3
S79N	-2.7	G51W	-0.5
S80Y	-0.5	G51V	4.6
S80P	-0.8	K52E	0.4
V81A	-2.4	K52R	-0.6
L82P	-5.4	K52N	0.4
Q85K	-0.4	G53C	2.9
R86W	3.6	D54Y	2.2
D89G	3.1	D54V	7.7
G90V	4.6	S55P	-0.8
P91H	-1.6	E56A	5.3
S93N	-2.7	E56V	7.7
P96Q	-1.9	P60T	0.9
S97T	0.1	P60H	-1.6
S97P	-0.8	P60L	5.4
S98T	0.1	D62Y	2.2
S98P	-0.8	P64T	0.9
Q99H	0.3	E66V	7.7
Q99L	7.3	E66D	0.0
D100Y	2.2	A71T	-2.5
S101Y	-0.5	C73R	-7.0
S101T	0.1	P74T	0.9
S103Y	-0.5	I75T	-5.2
P109T	0.9	I76N	-8.0
L111P	-5.4	V78D	-7.7
Y112C	3.8	S79G	0.4
Y112H	-1.9	S80Y	-0.5
D113V	7.7	S80P	-0.8
D113E	0.0	L82I	0.7
R114H	1.3	Q85K	-0.4
R115M	6.4	P87L	5.4
S116G	0.4	D89G	3.1
I117V	-0.3	G90V	4.6
I117T	-5.2	S93C	3.3

F118I	1.7	P96Q	-1.9
F118L	1.0	S98P	-0.8
D119Y	2.2	Q99H	0.3
V121M	-2.3	D100Y	2.2
V121E	-7.7	S101Y	-0.5
Q123K	-0.4	S103Y	-0.5
N125I	8.0	P109T	0.9
Q127H	0.3	R110G	4.1
E130D	0.0	Y112N	-2.2
S131C	3.3	D113E	0.0
S131G	0.4	D113V	7.7
Q137H	0.3	R115S	3.7
R138M	6.4	R115M	6.4
R138W	3.6	I117T	-5.2
K140E	0.4	I117V	-0.3
K140N	0.4	F118I	1.7
K140M	5.8	F118L	1.0
K141R	-0.6	D119Y	2.2
L143Q	-7.3	V121M	-2.3
D145G	3.1	V121E	-7.7
D145V	7.7	V121A	-2.4
S146C	3.3	Q123H	0.3
E147G	3.1	Q123K	-0.4
K149E	0.4	N125I	8.0
P151Q	-1.9	Q127L	7.3
E152G	3.1	Q127P	1.9
T153S	-0.1	L129Q	-7.3
T153I	5.2	E130G	3.1
G154V	4.6	E130D	0.0
K155R	-0.6	S131G	0.4
T156S	-0.1	L133P	-5.4
C157R	-7.0	Q137R	-1.0
L158Q	-7.3	Q137H	0.3
K160E	0.4	R138M	6.4
M162L	1.9	R138S	3.7
L163P	-5.4	K140R	-0.6
N164K	-0.4	K141E	0.4
N164S	2.7	K141R	-0.6
L165P	-5.4	L143Q	-7.3
H166R	-1.3	L143P	-5.4
H166L	7.0	D145Y	2.2
N167K	-0.4	F148L	1.0
N170K	-0.4	K149E	0.4
D171V	7.7	D150Y	2.2
T172A	2.5	P151Q	-1.9

T172S	-0.1	E152G	3.1
I173N	-8.0	E152D	0.0
L176P	-5.4	T153I	5.2
L177Q	-7.3	G154V	4.6
L177P	-5.4	K155N	0.4
D178Y	2.2	K155T	3.2
D178V	7.7	L158Q	-7.3
V179I	0.3	M162T	-2.6
V179A	-2.4	M162L	1.9
T183A	2.5	L163P	-5.4
S185G	0.4	N164S	2.7
L186P	-5.4	N164K	-0.4
K187R	-0.6	L165P	-5.4
F189L	1.0	N167D	0.0
V190A	-2.4	N167S	2.7
V190D	-7.7	G168V	4.6
N191D	0.0	Q169P	1.9
N191S	2.7	Q169H	0.3
Y194H	-1.9	D171Y	2.2
Y194N	-2.2	D171V	7.7
T195A	2.5	T172S	-0.1
Y198C	3.8	T172A	2.5
K200D	0.4	I173N	-8.0
K200C	6.4	L175M	-1.9
Q202L	7.3	L177Q	-7.3
L205Q	-7.3	L177P	-5.4
L205R	-8.3	D178Y	2.2
I207T	-5.2	D178V	7.7
I209N	-8.0	V179I	0.3
I209T	-5.2	V179A	-2.4
I209F	-1.7	K182R	-0.6
E210G	3.1	K182N	0.4
E210D	0.0	T183A	2.5
R211W	3.6	D184Y	2.2
R211L	8.3	D184G	3.1
T215S	-0.1	L186P	-5.4
L216Q	-7.3	K187R	-0.6
V217M	-2.3	F189I	1.7
V217E	-7.7	V190D	-7.7
V217A	-2.4	V190A	-2.4
T218S	-0.1	N191D	0.0
L219H	-7.0	N191S	2.7
L220F	-1.0	Y194N	-2.2
V221E	-7.7	Y194H	-1.9
E222D	0.0	T195A	2.5

N223D	0.0	D196Y	2.2
D226G	3.1	S197G	0.4
V227A	-2.4	Y198H	-1.9
Q228K	-0.4	Y198C	3.8
Q228L	7.3	G201D	-3.1
Q228H	0.3	G201C	2.9
A231T	-2.5	Q202L	7.3
N232I	8.0	Q202H	0.3
G233E	-3.1	L205Q	-7.3
F235S	-3.6	I207T	-5.2
F236L	1.0	I207F	-1.7
K237N	0.4	I209T	-5.2
K237E	0.4	I209F	-1.7
K240N	0.4	E210G	3.1
G241V	4.6	E210D	0.0
P243H	-1.6	R211W	3.6
P243L	5.4	R211L	8.3
G244C	2.9	R212L	8.3
F245S	-3.6	N213Y	2.2
F247S	-3.6	M214L	1.9
G248C	2.9	M214I	2.6
E249V	7.7	M214T	-2.6
E249G	3.1	L216Q	-7.3
P251H	-1.6	L216P	-5.4
L252Q	-7.3	V217E	-7.7
L252P	-5.4	V217A	-2.4
S253T	0.1	T218S	-0.1
S253Y	-0.5	L219I	0.7
A255T	-2.5	L219H	-7.0
C257R	-7.0	L220S	-4.6
T258A	2.5	L220F	-1.0
N259I	8.0	V221E	-7.7
N259D	0.0	E222D	0.0
N259Y	2.2	N223D	0.0
Q260R	-1.0	N223I	8.0
Q260L	7.3	G224V	4.6
L261Q	-7.3	D226G	3.1
V264E	-7.7	Q228R	-1.0
L267M	-1.9	Q228K	-0.4
L267P	-5.4	D234Y	2.2
N270S	2.7	F235S	-3.6
W272R	-3.6	F235V	1.4
W272L	4.7	F236L	1.0
D276Y	2.2	F236I	1.7
G284C	2.9	F236Y	-4.1

N285I	8.0	F236S	-3.6
N285D	0.0	K237E	0.4
V287E	-7.7	K237R	-0.6
L288P	-5.4	K237N	0.4
H289N	-0.3	T239S	-0.1
H289R	-1.3	G241V	4.6
L291P	-5.4	R242G	4.1
V292G	-4.6	R242M	6.4
V292E	-7.7	P243L	5.4
E293D	0.0	G244C	2.9
E293G	3.1	F245S	-3.6
V294E	-7.7	Y246H	-1.9
V294A	-2.4	F247S	-3.6
A295T	-2.5	G248C	2.9
D296V	7.7	E249G	3.1
D296E	0.0	E249V	7.7
N301I	8.0	L250P	-5.4
N301D	0.0	L252M	-1.9
N301S	2.7	L252Q	-7.3
T302A	2.5	L252P	-5.4
F304S	-3.6	S253T	0.1
V305M	-2.3	S253Y	-0.5
V305A	-2.4	L254M	-1.9
T306S	-0.1	C257R	-7.0
S307R	-3.7	T258A	2.5
S307G	0.4	Q260R	-1.0
M308T	-2.6	Q260L	7.3
Y309H	-1.9	L261P	-5.4
Y309C	3.8	I263T	-5.2
N310I	8.0	V264E	-7.7
E311G	3.1	F266L	1.0
I312N	-8.0	L267M	-1.9
I312T	-5.2	L267P	-5.4
L313S	-4.6	Q269R	-1.0
I314V	-0.3	Q269H	0.3
I314N	-8.0	N270S	2.7
G316W	-0.5	S271Y	-0.5
L319P	-5.4	W272R	-3.6
H320P	1.6	W272L	4.7
H320L	7.0	D276Y	2.2
T322M	2.6	D276V	7.7
L323P	-5.4	S278G	0.4
K324R	-0.6	D281Y	2.2
L325P	-5.4	D281G	3.1
L325Q	-7.3	G284C	2.9

I328V	-0.3	N285I	8.0
I328T	-5.2	V287E	-7.7
T329A	2.5	V287A	-2.4
N330Y	2.2	L288P	-5.4
N330I	8.0	H289N	-0.3
R331M	6.4	H289R	-1.3
R331S	3.7	L291M	-1.9
K332N	0.4	L291P	-5.4
G333W	-0.5	E293G	3.1
G333V	4.6	V294E	-7.7
T335P	-0.9	V294A	-2.4
T335A	2.5	A295V	2.4
T335M	2.6	A295T	-2.5
L337P	-5.4	D296V	7.7
L337Q	-7.3	N297I	8.0
L339Q	-7.3	T298A	2.5
L339P	-5.4	D300Y	2.2
S343R	-3.7	N301D	0.0
G344R	-4.1	N301I	8.0
G344V	4.6	T302A	2.5
K345R	-0.6	K303M	5.8
I346F	-1.7	V305E	-7.7
G347R	-4.1	T306S	-0.1
V348A	-2.4	S307G	0.4
L349S	-4.6	M308T	-2.6
L349F	-1.0	Y309C	3.8
I352T	-5.2	N310D	0.0
I352V	-0.3	N310I	8.0
L353I	0.7	I312N	-8.0
L353P	-5.4	I314N	-8.0
Q354H	0.3	I314T	-5.2
Q354R	-1.0	I314V	-0.3
R355S	3.7	G316W	-0.5
E356G	3.1	L319P	-5.4
H358L	7.0	H320R	-1.3
H358R	-1.3	P321H	-1.6
E359G	3.1	P321T	0.9
P360H	-1.6	L323P	-5.4
E361G	3.1	K324R	-0.6
H364R	-1.3	L325P	-5.4
L365I	0.7	E327G	3.1
R367M	6.4	I328N	-8.0
E371K	-0.4	T329A	2.5
W372R	-3.6	N330I	8.0
W372L	4.7	R331M	6.4

G375E	-3.1	R331S	3.7
P376S	0.8	K332N	0.4
P376L	5.4	G333W	-0.5
V377A	-2.4	G333V	4.6
H378R	-1.3	L334P	-5.4
S380Y	-0.5	T335P	-0.9
S380P	-0.8	L337Q	-7.3
Y382H	-1.9	A338V	2.4
L384M	-1.9	L339Q	-7.3
S385Y	-0.5	L339P	-5.4
C386Y	-3.8	A340T	-2.5
I387T	-5.2	S343R	-3.7
D388V	7.7	G344V	4.6
E391G	3.1	K345R	-0.6
K392E	0.4	I346F	-1.7
K392R	-0.6	G347R	-4.1
N393S	2.7	G347V	4.6
N393I	8.0	L349S	-4.6
S394P	-0.8	L349F	-1.0
V395A	-2.4	I352T	-5.2
L396Q	-7.3	L353P	-5.4
E397G	3.1	L353I	0.7
E397D	0.0	Q354R	-1.0
V398A	-2.4	Q354K	-0.4
I399N	-8.0	Q354H	0.3
S402G	0.4	R355W	3.6
S402C	3.3	R355S	3.7
S403G	0.4	E356G	3.1
S404R	-3.7	P360H	-1.6
S404G	0.4	E361V	7.7
E405D	0.0	R363L	8.3
E405V	7.7	H364R	-1.3
T406S	-0.1	R367M	6.4
N408I	8.0	F369L	1.0
N408S	2.7	F369S	-3.6
H410L	7.0	E371K	-0.4
D411N	0.0	W372L	4.7
D411V	7.7	G375W	-0.5
M412L	1.9	G375E	-3.1
M412T	-2.6	P376S	0.8
L413P	-5.4	P376L	5.4
L414I	0.7	V377A	-2.4
L414H	-7.0	V377E	-7.7
L414P	-5.4	S379Y	-0.5
L414F	-1.0	S379P	-0.8

V415A	-2.4	S380P	-0.8
V415E	-7.7	S380Y	-0.5
E416D	0.0	Y382C	3.8
P417H	-1.6	D383Y	2.2
P417S	0.8	D383G	3.1
N419Y	2.2	D383V	7.7
L421H	-7.0	L384M	-1.9
L422I	0.7	S385Y	-0.5
Q423H	0.3	S385P	-0.8
Q423R	-1.0	C386Y	-3.8
D424Y	2.2	C386R	-7.0
K425E	0.4	D388Y	2.2
D427G	3.1	D388V	7.7
D427Y	2.2	E391G	3.1
D427V	7.7	K392R	-0.6
K431E	0.4	K392N	0.4
I433I	0.0	N393I	8.0
I433L	-0.7	S394P	-0.8
Y435L	5.1	V395A	-2.4
F436D	-6.3	L396M	-1.9
N437I	8.0	L396Q	-7.3
N437L	7.3	E397D	0.0
N437S	2.7	V398A	-2.4
F439D	-6.3	I399N	-8.0
M445K	-5.8	I399T	-5.2
M445R	-6.4	S404G	0.4
I446N	-8.0	E405V	7.7
I446L	-0.7	E405D	0.0
I446F	-1.7	T406S	-0.1
F448L	1.0	N408I	8.0
Y453H	-1.9	H410N	-0.3
R455Q	1.0	D411Y	2.2
R455L	8.3	M412T	-2.6
V457A	-2.4	M412L	1.9
L460S	-4.6	L413P	-5.4
P461S	0.8	L414H	-7.0
P461L	5.4	L414P	-5.4
K464M	5.8	E416D	0.0
L465P	-5.4	P417H	-1.6
N467T	2.8	L418F	-1.0
T468A	2.5	N419Y	2.2
V469I	0.3	R420L	8.3
D471G	3.1	L421H	-7.0
D471Y	2.2	L422I	0.7
F473L	1.0	Q423R	-1.0

R474L	8.3	Q423H	0.3
V475D	-7.7	D424Y	2.2
V475A	-2.4	K425E	0.4
T476S	-0.1	K425M	5.8
T476A	2.5	W426R	-3.6
G477R	-4.1	W426C	3.4
G477E	-3.1	D427V	7.7
G477V	4.6	D427G	3.1
I479V	-0.3	D427Y	2.2
S481F	3.6	K431E	0.4
S481P	-0.8	I433T	-5.2
V482A	-2.4	F434L	1.0
S483P	-0.8	F434I	1.7
S483T	0.1	F436L	1.0
G484V	4.6	F438I	1.7
G485V	4.6	F438L	1.0
V486D	-7.7	F438S	-3.6
Y487C	3.8	V440D	-7.7
F488S	-3.6	V440A	-2.4
F489L	1.0	L443S	-4.6
F489S	-3.6	Y444H	-1.9
R491L	8.3	M445L	1.9
G492V	4.6	M445K	-5.8
I493V	-0.3	I446N	-8.0
F496L	1.0	I446L	-0.7
F496S	-3.6	I446F	-1.7
L497M	-1.9	I447F	-1.7
L497P	-5.4	F448L	1.0
R499G	4.1	Y454H	-1.9
S502Y	-0.5	R455Q	1.0
S502P	-0.8	V457A	-2.4
L503H	-7.0	P461H	-1.6
K504N	0.4	K464M	5.8
S505G	0.4	T468A	2.5
L506S	-4.6	G470W	-0.5
F507Y	-4.1	G470V	4.6
F507S	-3.6	D471Y	2.2
V508E	-7.7	F473L	1.0
V508A	-2.4	R474L	8.3
D509G	3.1	V475D	-7.7
D509V	7.7	T476A	2.5
Y511H	-1.9	G477V	4.6
Y511F	4.1	E478G	3.1
S512G	0.4	I479V	-0.3
E513G	3.1	S481P	-0.8

E513D	0.0	S481Y	-0.5
I514M	-2.6	S481F	3.6
I514V	-0.3	V482A	-2.4
L515P	-5.4	S483T	0.1
F516L	1.0	S483P	-0.8
F517I	1.7	G484V	4.6
F517L	1.0	G485V	4.6
Q519L	7.3	V486D	-7.7
Q519R	-1.0	Y487N	-2.2
L521P	-5.4	F488Y	-4.1
F522S	-3.6	F489L	1.0
M523R	-6.4	R491L	8.3
M523T	-2.6	G492W	-0.5
L524Q	-7.3	G492V	4.6
L524P	-5.4	I493V	-0.3
S526P	-0.8	Q494K	-0.4
V527E	-7.7	F496L	1.0
V527A	-2.4	L497P	-5.4
V528A	-2.4	L497M	-1.9
L529Q	-7.3	R499G	4.1
L529P	-5.4	R500L	8.3
Y530H	-1.9	P501Q	-1.9
Y530C	3.8	S502P	-0.8
S532G	0.4	S502Y	-0.5
Q533K	-0.4	L503H	-7.0
Q533R	-1.0	K504N	0.4
K535M	5.8	F507Y	-4.1
K535N	0.4	F507S	-3.6
K535E	0.4	V508A	-2.4
K535R	-0.6	V508E	-7.7
E536G	3.1	D509G	3.1
Y537F	4.1	D509Y	2.2
V538A	-2.4	S510G	0.4
M541K	-5.8	Y511H	-1.9
M541I	2.6	S512G	0.4
F543S	-3.6	S512R	-3.7
S544P	-0.8	E513G	3.1
L545Q	-7.3	I514V	-0.3
W549R	-3.6	L515P	-5.4
W549C	3.4	F516I	1.7
T550A	2.5	F516L	1.0
M552V	2.3	F517I	1.7
L553P	-5.4	F517L	1.0
Y554N	-2.2	Q519R	-1.0
Y554H	-1.9	Q519L	7.3

Y554C	3.8	S520P	-0.8
Y555C	3.8	L521P	-5.4
Y555H	-1.9	F522S	-3.6
Y555F	4.1	M523K	-5.8
T556A	2.5	M523R	-6.4
F559L	1.0	L524P	-5.4
Q560K	-0.4	V525A	-2.4
Q560R	-1.0	S526Y	-0.5
M562T	-2.6	S526P	-0.8
M562V	2.3	V527A	-2.4
M562L	1.9	V527E	-7.7
I564T	-5.2	V528A	-2.4
Y565N	-2.2	L529P	-5.4
Y565C	3.8	L529Q	-7.3
Y565H	-1.9	Q533H	0.3
V567D	-7.7	Q533K	-0.4
V567A	-2.4	Q533R	-1.0
M568T	-2.6	Q533L	7.3
M568I	2.6	K535E	0.4
M568V	2.3	K535R	-0.6
I569V	-0.3	Y537S	0.5
I569T	-5.2	Y537C	3.8
E570G	3.1	V538A	-2.4
K571E	0.4	V538E	-7.7
K571R	-0.6	F543L	1.0
M572T	-2.6	F543S	-3.6
M572V	2.3	S544Y	-0.5
M572L	1.9	S544P	-0.8
I573V	-0.3	S544T	0.1
L574H	-7.0	M547T	-2.6
L574P	-5.4	M547V	2.3
D576Y	2.2	M547I	2.6
D576V	7.7	W549R	-3.6
D576G	3.1	W549C	3.4
L577M	-1.9	T550A	2.5
L577P	-5.4	N551S	2.7
R579Q	1.0	L553P	-5.4
F580I	1.7	Y554N	-2.2
F580S	-3.6	Y554H	-1.9
F580L	1.0	G558E	-3.1
M581K	-5.8	F559L	1.0
M581T	-2.6	Q560K	-0.4
M581V	2.3	M562L	1.9
V583I	0.3	M562I	2.6
V583D	-7.7	M562T	-2.6

Y584H	-1.9	M562V	2.3
Y584C	3.8	I564N	-8.0
L585P	-5.4	Y565N	-2.2
F587Y	-4.1	Y565C	3.8
F587S	-3.6	V567D	-7.7
L588S	-4.6	V567A	-2.4
F589S	-3.6	M568T	-2.6
F589L	1.0	M568V	2.3
F591S	-3.6	M568R	-6.4
F591L	1.0	M568I	2.6
S592Y	-0.5	I569T	-5.2
S592P	-0.8	I569V	-0.3
T593S	-0.1	I569F	-1.7
V595E	-7.7	E570G	3.1
V595A	-2.4	M572L	1.9
V596M	-2.3	I573V	-0.3
V596A	-2.4	L574P	-5.4
T597A	2.5	R575G	4.1
T597S	-0.1	D576V	7.7
L598P	-5.4	D576G	3.1
I599T	-5.2	D576Y	2.2
I599F	-1.7	L577M	-1.9
E600G	3.1	L577P	-5.4
E600V	7.7	R579Q	1.0
E600D	0.0	F580L	1.0
D601G	3.1	F580S	-3.6
G602V	4.6	M581T	-2.6
K603N	0.4	M581K	-5.8
N604Y	2.2	M581V	2.3
N604D	0.0	V583I	0.3
N604S	2.7	V583D	-7.7
N605S	2.7	V583A	-2.4
L607P	-5.4	Y584H	-1.9
P608H	-1.6	Y584C	3.8
M609L	1.9	L585P	-5.4
M609I	2.6	F587L	1.0
M609K	-5.8	F587I	1.7
E610G	3.1	F587Y	-4.1
P613Q	-1.9	F587S	-3.6
H614R	-1.3	L588S	-4.6
C616R	-7.0	F589L	1.0
G618V	4.6	F589Y	-4.1
S619Y	-0.5	F591L	1.0
S619P	-0.8	F591S	-3.6
C621R	-7.0	S592Y	-0.5

K622E	0.4	V595E	-7.7
K622R	-0.6	V595A	-2.4
P623Q	-1.9	V596E	-7.7
G624C	2.9	V596M	-2.3
N625S	2.7	T597S	-0.1
S626P	-0.8	T597A	2.5
Y627H	-1.9	L598P	-5.4
Y627F	4.1	I599T	-5.2
N628D	0.0	I599F	-1.7
N628S	2.7	E600G	3.1
N628I	8.0	E600D	0.0
S629G	0.4	D601G	3.1
L630Q	-7.3	D601E	0.0
L630P	-5.4	G602V	4.6
Y631H	-1.9	K603R	-0.6
T633A	2.5	K603N	0.4
T633S	-0.1	N604D	0.0
C634R	-7.0	N605S	2.7
L635M	-1.9	S606Y	-0.5
L635P	-5.4	S606P	-0.8
E636G	3.1	L607M	-1.9
F638I	1.7	L607Q	-7.3
F638S	-3.6	P608H	-1.6
K639R	-0.6	M609I	2.6
F640I	1.7	M609L	1.9
F640Y	-4.1	E610V	7.7
F640S	-3.6	S611Y	-0.5
T641A	2.5	P613Q	-1.9
I642V	-0.3	H614R	-1.3
I642F	-1.7	K615E	0.4
G643C	2.9	K615R	-0.6
G643S	-0.4	C616R	-7.0
M644T	-2.6	G618W	-0.5
M644V	2.3	G618V	4.6
D646V	7.7	S619P	-0.8
L647M	-1.9	S619Y	-0.5
L647Q	-7.3	K622E	0.4
L647P	-5.4	P623Q	-1.9
E648G	3.1	G624C	2.9
F649Y	-4.1	N625I	8.0
T650A	2.5	S626Y	-0.5
E651G	3.1	S626P	-0.8
E651V	7.7	S626T	0.1
E651D	0.0	N628D	0.0
N652Y	2.2	N628S	2.7

N652I	8.0	L630Q	-7.3
Y653F	4.1	L630P	-5.4
D654G	3.1	Y631H	-1.9
D654V	7.7	Y631C	3.8
F655L	1.0	S632Y	-0.5
K656M	5.8	S632P	-0.8
K656E	0.4	T633S	-0.1
K656R	-0.6	C634R	-7.0
V658D	-7.7	L635M	-1.9
V658A	-2.4	L635Q	-7.3
F659L	1.0	L635P	-5.4
I661T	-5.2	E636G	3.1
L662Q	-7.3	L637R	-8.3
L663S	-4.6	F638L	1.0
L664P	-5.4	F638Y	-4.1
Y666C	3.8	F638S	-3.6
V667A	-2.4	K639E	0.4
I668F	-1.7	K639R	-0.6
I668T	-5.2	F640L	1.0
L669H	-7.0	F640Y	-4.1
L669P	-5.4	F640S	-3.6
T670A	2.5	T641A	2.5
Y671H	-1.9	T641I	5.2
I672T	-5.2	I642V	-0.3
I672V	-0.3	I642N	-8.0
L673P	-5.4	I642T	-5.2
L675H	-7.0	I642F	-1.7
L675P	-5.4	G643S	-0.4
L675F	-1.0	G643C	2.9
N676D	0.0	M644K	-5.8
N676S	2.7	M644T	-2.6
M677T	-2.6	M644I	2.6
M677V	2.3	M644V	2.3
M677L	1.9	D646Y	2.2
L678H	-7.0	D646V	7.7
L678P	-5.4	L647M	-1.9
I679T	-5.2	L647Q	-7.3
L681H	-7.0	L647P	-5.4
L681P	-5.4	L647R	-8.3
M682T	-2.6	E648G	3.1
M682L	1.9	F649Y	-4.1
M682V	2.3	F649L	1.0
G683S	-0.4	F649S	-3.6
G683C	2.9	T650A	2.5
E684G	3.1	E651G	3.1

T685A	2.5	E651D	0.0
N687D	0.0	N652S	2.7
N687S	2.7	N652Y	2.2
N687I	8.0	N652I	8.0
I689T	-5.2	Y653H	-1.9
I689V	-0.3	Y653F	4.1
A690T	-2.5	D654G	3.1
Q691R	-1.0	D654Y	2.2
E692G	3.1	F655L	1.0
S693G	0.4	F655V	1.4
S693C	3.3	K656R	-0.6
K694R	-0.6	K656M	5.8
N695H	0.3	V658A	-2.4
N695S	2.7	V658D	-7.7
N695Y	2.2	F659L	1.0
N695D	0.0	F659S	-3.6
N695I	8.0	I660T	-5.2
I696T	-5.2	I660V	-0.3
I696V	-0.3	I661T	-5.2
W697R	-3.6	I661N	-8.0
K698M	5.8	L662M	-1.9
K698R	-0.6	L662P	-5.4
L699P	-5.4	L662Q	-7.3
Q700L	7.3	L662R	-8.3
Q700R	-1.0	L663S	-4.6
R701G	4.1	L664P	-5.4
R701I	9.0	Y666H	-1.9
I703F	-1.7	Y666C	3.8
I703N	-8.0	V667A	-2.4
I703T	-5.2	I668F	-1.7
T704A	2.5	I668T	-5.2
I705T	-5.2	I668V	-0.3
I705V	-0.3	L669I	0.7
L706M	-1.9	L669H	-7.0
L706P	-5.4	L669P	-5.4
D707Y	2.2	T670A	2.5
E709V	7.7	T670I	5.2
E709D	0.0	Y671H	-1.9
E709G	3.1	I672V	-0.3
K710E	0.4	I672T	-5.2
K710M	5.8	L673I	0.7
K710N	0.4	L673P	-5.4
K710R	-0.6	L674P	-5.4
F712L	1.0	L675P	-5.4
L713Q	-7.3	N676D	0.0

L713P	-5.4	N676S	2.7
K714E	0.4	M677V	2.3
M716V	2.3	M677K	-5.8
M716R	-6.4	M677T	-2.6
R717G	4.1	M677L	1.9
R717S	3.7	L678H	-7.0
K718E	0.4	L678P	-5.4
K718N	0.4	I679T	-5.2
A719T	-2.5	I679L	-0.7
F720L	1.0	L681I	0.7
F720S	-3.6	L681H	-7.0
R721S	3.7	L681P	-5.4
R721H	1.3	M682T	-2.6
G723D	-3.1	M682V	2.3
G723C	2.9	M682L	1.9
K724E	0.4	G683C	2.9
L725P	-5.4	E684G	3.1
L726P	-5.4	E684D	0.0
Q727H	0.3	T685A	2.5
Q727R	-1.0	N687D	0.0
V728A	-2.4	N687S	2.7
V728E	-7.7	N687I	8.0
G729V	4.6	I689V	-0.3
F730S	-3.6	Q691R	-1.0
P732T	0.9	E692D	0.0
P732H	-1.6	E692G	3.1
D733Y	2.2	S693C	3.3
D733G	3.1	S693G	0.4
D733V	7.7	K694N	0.4
G734D	-3.1	K694R	-0.6
D736Y	2.2	N695Y	2.2
D737G	3.1	N695H	0.3
D737V	7.7	N695D	0.0
R739L	8.3	N695S	2.7
W740L	4.7	N695I	8.0
C741R	-7.0	I696V	-0.3
C741S	-3.3	I696N	-8.0
F742L	1.0	I696T	-5.2
D745G	3.1	W697R	-3.6
D745Y	2.2	W697L	4.7
E746D	0.0	K698R	-0.6
E746G	3.1	K698E	0.4
N748I	8.0	L699P	-5.4
W749R	-3.6	Q700L	7.3
T750A	2.5	Q700H	0.3

W752R	-3.6	R701G	4.1
T754A	2.5	R701I	9.0
N755K	-0.4	A702V	2.4
V756E	-7.7	I703N	-8.0
V756A	-2.4	I703T	-5.2
G757C	2.9	I703F	-1.7
I758N	-8.0	I705T	-5.2
I759V	-0.3	I705F	-1.7
I759N	-8.0	L706M	-1.9
I759T	-5.2	L706P	-5.4
I759F	-1.7	D707G	3.1
N760S	2.7	D707Y	2.2
N760D	0.0	E709V	7.7
N760I	8.0	E709D	0.0
E761V	7.7	K710M	5.8
E761D	0.0	K710N	0.4
D762G	3.1	F712L	1.0
C766Y	-3.8	F712S	-3.6
C766R	-7.0	L713P	-5.4
G768C	2.9	C715Y	-3.8
K770E	0.4	M716I	2.6
R771H	1.3	M716V	2.3
L773Q	-7.3	R717S	3.7
L773P	-5.4	K718N	0.4
S776P	-0.8	R721S	3.7
R778M	6.4	S722Y	-0.5
S779T	0.1	S722P	-0.8
G780D	-3.1	G723D	-3.1
V782A	-2.4	G723C	2.9
V782D	-7.7	K724M	5.8
G784R	-4.1	L726P	-5.4
R785S	3.7	Q727H	0.3
N786D	0.0	V728E	-7.7
W787L	4.7	G729V	4.6
W787R	-3.6	F730I	1.7
N789D	0.0	F730S	-3.6
P794T	0.9	P732T	0.9
L795P	-5.4	P732H	-1.6
R797G	4.1	D733Y	2.2
D798Y	2.2	D733V	7.7
T801A	2.5	G734D	-3.1
D803G	3.1	G734C	2.9
R804I	9.0	G734S	-0.4
H805R	-1.3	K735N	0.4
A806T	-2.5	D736G	3.1

T807S	-0.1	D736Y	2.2
Q808R	-1.0	D737Y	2.2
Q809H	0.3	D737G	3.1
Q809R	-1.0	D737V	7.7
L814P	-5.4	Y738C	3.8
K815R	-0.6	W740L	4.7
Y817C	3.8	F742S	-3.6
T818A	2.5	F742L	1.0
S820P	-0.8	D745G	3.1
K822R	-0.6	D745Y	2.2
D825G	3.1	E746D	0.0
D825V	7.7	N748S	2.7
E827D	0.0	N748D	0.0
F829L	1.0	W749L	4.7
K830R	-0.6	W749R	-3.6
D831Y	2.2	W749C	3.4
S832Y	-0.5	T750A	2.5
M833T	-2.6	W752L	4.7
V834D	-7.7	W752C	3.4
P835T	0.9	N753S	2.7
E837D	0.0	T754A	2.5
E837G	3.1	N755Y	2.2
		G757C	2.9
		I758N	-8.0
		I759T	-5.2
		I759V	-0.3
		N760D	0.0
		N760S	2.7
		E761V	7.7
		E761D	0.0
		D762Y	2.2
		P763Q	-1.9
		P763T	0.9
		C766Y	-3.8
		E767G	3.1
		E767V	7.7
		G768C	2.9
		T772A	2.5
		L777M	-1.9
		R778M	6.4
		S779T	0.1
		S779L	4.6
		G780D	-3.1
		R781L	8.3
		R781Q	1.0

		V782D	-7.7
		V782A	-2.4
		G784V	4.6
		R785G	4.1
		R785I	9.0
		R785S	3.7
		N786D	0.0
		W787L	4.7
		W787C	3.4
		K788N	0.4
		N789D	0.0
		F790I	1.7
		L792P	-5.4
		P794T	0.9
		L795I	0.7
		L795P	-5.4
		R797S	3.7
		T801A	2.5
		R802L	8.3
		D803G	3.1
		R804I	9.0
		A806T	-2.5
		T807A	2.5
		T807S	-0.1
		Q808K	-0.4
		Q808R	-1.0
		Q809R	-1.0
		Q809H	0.3
		V812A	-2.4
		Q813K	-0.4
		Q813R	-1.0
		Y817C	3.8
		T818A	2.5
		S820P	-0.8
		L821I	0.7
		K822R	-0.6
		E824D	0.0
		E827G	3.1
		E827D	0.0
		F829L	1.0
		K830R	-0.6
		K830N	0.4
		D831Y	2.2
		S832Y	-0.5
		M833V	2.3

		M833I	2.6
		V834D	-7.7
		V834A	-2.4
		P835Q	-1.9
		P835T	0.9
		E837D	0.0

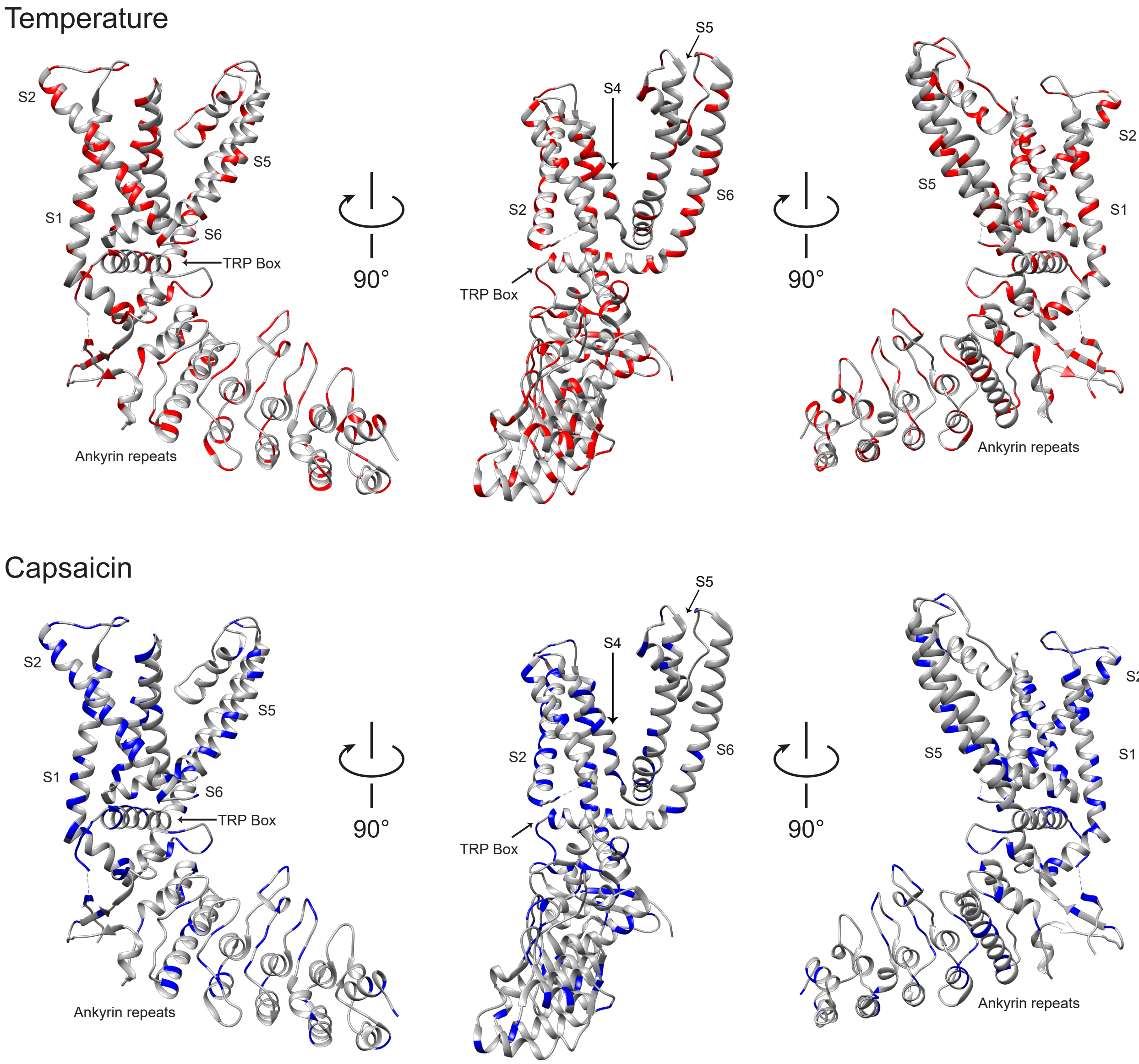

Supplementary Figure S1. Illustration of temperature 'functional' and capsaicin 'functional' mutations

Temperature 'functional' (top panel) and capsaicin 'functional' (bottom panel) mutations localized onto the apo high-resolution structure of TRPV1. Temperature mutations are in red. Capsaicin mutations are in blue.
